# Eosinophilia Associated With CD3^−^CD4^+^ T Cells: Characterization and Outcome of a Single-Center Cohort of 26 Patients

**DOI:** 10.3389/fimmu.2020.01765

**Published:** 2020-08-11

**Authors:** Caroline Carpentier, Sylvain Verbanck, Liliane Schandené, Pierre Heimann, Anne-Laure Trépant, Elie Cogan, Florence Roufosse

**Affiliations:** ^1^Department of Internal Medicine, Hôpital Erasme, Université Libre de Bruxelles, Brussels, Belgium; ^2^Laboratory of Immunobiology, Hôpital Erasme, Université Libre de Bruxelles, Brussels, Belgium; ^3^Department of Medical Genetics, Hôpital Erasme, Université Libre de Bruxelles, Brussels, Belgium; ^4^Department of Pathology, Hôpital Erasme, Université Libre de Bruxelles, Brussels, Belgium; ^5^Institute for Medical Immunology, Université Libre de Bruxelles, Brussels, Belgium

**Keywords:** lymphocytic variant hypereosinophilic syndrome, CD3^−^CD4^+^ T cells, interleukin-5, TARC (CCL17), fasciitis, interferon-alpha, anti-interleukin-5, T cell lymphoma

## Abstract

**Background:** Lymphocytic variant hypereosinophilic syndrome is characterized by marked over-production of eosinophilopoietic factor(s) by dysregulated T cells leading to eosinophil expansion. In most cases, these T cells are clonal and express a CD3^−^CD4^+^ phenotype. As this is a rare disorder, presenting manifestations, disease course, treatment responses, and outcome are not well-characterized.

**Materials and Methods:** In this retrospective single-center observational study, we reviewed medical files of all patients with persistent hypereosinophilia seen between 1994 and 2019 in whom CD3^−^CD4^+^ T cells were detected. Data collection included clinical and biological findings at presentation, treatment responses, disease course, and serial CD3^−^CD4^+^ T cell counts.

**Results:** Our cohort comprises 26 patients, including 2 with hypereosinophilia of undetermined significance. All 24 symptomatic patients had cutaneous lesions and/or angioedema, and fasciitis was present in several cases. The aberrant T cell subset represented 2% or less total lymphocytes in 11 subjects. TCR gene rearrangement patterns on whole blood were polyclonal in these cases, while they all had serum CCL17/TARC levels above 1,500 pg/ml. Disease manifestations were mild and did not require maintenance therapy in roughly one third of the cohort, while two thirds required long-term oral corticosteroids and/or second-line agents. Among these, interferon-alpha was the most effective treatment option with a response observed in 8/8 patients, one of whom was cured of disease. Treatment had to be interrupted in most cases however due to poor tolerance and/or development of secondary resistance. Anti-interleukin-5 antibodies reduced blood eosinophilia in 5/5 patients, but clinical responses were disappointing. A sub-group of 5 patients had severe treatment-refractory disease, and experienced significant disease- and treatment-related morbidity and mortality, including progression to T cell lymphoma in three.

**Conclusions:** This retrospective longitudinal analysis of the largest monocentric cohort of CD3^−^CD4^+^ T cell associated lymphocytic variant hypereosinophilic syndrome published so far provides clinicians confronted with this rare disorder with relevant new data on patient presentation and outcome that should help tailor therapy and follow-up to different levels of disease severity. It highlights the need for novel therapeutic options, especially for the subset of patients with severe treatment-refractory disease. Future research efforts should be made toward understanding CD3^−^CD4^+^ T cell biology in order to develop new treatments that target primary pathogenic mechanisms.

## Introduction

The term hypereosinophilic syndrome (HES) has been used since 1968 to describe and classify patients with persistently elevated blood and tissue eosinophil levels resulting in organ damage and dysfunction (1). The first published patient series revealed that extremely diverse clinical presentations and outcomes could be observed within this entity, with some patients developing rapidly fatal heart disease due to subendocardial fibrosis, while others suffer minimal disease manifestations such as isolated urticaria or eczema (2). Progression to acute myeloid or eosinophilic leukemia in some cases, and more protracted development of T cell lymphoma in others, represented early indices that profoundly different underlying pathogenic mechanisms, involving different cell types (i.e., myeloid vs. lymphoid lineages) were likely to be the main reason for this clinical heterogeneity. Since then, distinct underlying pathogenic mechanisms have been described in patient subsets, and definitions and classifications of eosinophilic conditions have been revised on this basis.

According to the International Cooperative Working Group on Eosinophilic Disorders (ICOG-EO), hypereosinophilia (HE) is now defined on the basis of blood eosinophilia above 1.5 G/L on at least two occasions, and/or tissue eosinophilia that is judged excessive by an experimented pathologist (3). Diagnosis of HES requires that HE be present, in association with organ damage that cannot be explained by mechanisms other than eosinophil-mediated toxicity. A HES can therefore develop in the setting of chronic HE of any cause, provided it results in end-organ damage. Patients with HES are further sub-divided into 3 groups according to mechanisms underlying eosinophil expansion: (1) neoplastic HES (HES_N_), with a demonstrable cytogenetic/karyotypic perturbation resulting in clonal proliferation of eosinophils (most commonly F1P1L1/PDGFRA^+^ chronic eosinophilic leukemia) or increased blasts, (2) reactive HES (HES_R_), where increased/deregulated production of eosinophilopoietic factors drives polyclonal eosinophil expansion, and (3) idiopathic HES (HES_I_) when the mechanism driving eosinophilia remains elusive.

Lymphocytic (or lymphoid) variant HES (L-HES), first described 25 years ago by our group (4), is characterized by over-production of eosinophilopoietic cytokine(s) (most commonly IL-5) by deregulated T cells, and therefore falls in the HES_R_ category. Subsequent case reports and a recent multicenter patient series have described clinical presentations, disease course, and treatment responses in patients with L-HES (5–8). In the very large majority of cases where enhanced type-2 cytokine production is firmly established, the pathogenic T cells have been shown to display a TCRα/β^−^CD3^−^CD4^+^ surface phenotype and to be monoclonal. The current understanding is that this is an indolent lymphoproliferative disorder, with progression to T cell lymphoma occurring only in a minority of patients (9, 10). The primary cause driving T cell proliferation, activation, and enhanced IL-5 production in this disease remains unknown.

Twenty-five years after the first well-documented case of CD3^−^CD4^+^ T cell associated HES, with 25 additional patients seen in our academic hospital since then, we have analyzed retrospectively and in depth the clinical and biological features, treatment responses, disease course, and clinical outcome of this large single-center cohort.

## Patients and Methods

### Patient Identification and Recruitment

Our academic hospital has been a referral center for patients with HES for the past 25 years. Our approach to differential diagnosis of HE is a step-wise process, whereby classical underlying causes of HE such as drug allergy, helminthiasis, cancer and chronic inflammatory/auto-immune diseases are investigated thoroughly before undertaking more specific analyses aiming to detect HES variants. The latter analyses include molecular studies to rule out a myeloproliferative disorder; we search for the FIP1L1-PDGFRA fusion gene and eventually other mutations (i.e., BCR-ABL, PDGFRB, and FGFR1 rearrangements and JAK2 mutations) as warranted on the basis of disease presentation.

Since our first description of a functional link between CD3^−^CD4^+^ T cells and HE in a patient with HES reported in 1994 (4), our immunobiologist (LS) has tested different flow cytometry procedures allowing accurate detection of these cells. Since 2004, we have been using the same 5-color staining panel and acquisition strategy in all patients presenting with persistent unexplained HE in our center (Supplementary Table 1). When CD3^−^CD4^+^ cells are detected, patients are systematically re-tested for confirmation and in-depth assessment to rule out an underlying cutaneous or angioimmunoblastic T cell lymphoma are undertaken given the possible association of these hematological malignancies with this lymphocyte subset. Quantification of intracytoplasmic cytokines within CD3^−^CD4^+^ T cells are performed by flow cytometry as previously reported (7) to confirm over-production of type 2 cytokines and hence a functional role in disease. Briefly, peripheral blood mononuclear cells are stimulated *in vitro* with phorbol 12-myristate 13-acetate (PMA, 10 ng/ml) and A23187 ionophore (100 ng/ml) in presence of Brefeldin A (10 microg/ml) (all purchased from Sigma-Aldrich, Schnelldorf, Germany) for 6 h, surface-stained for CD3 and CD4 antigens, fixed and permeabilized (Fix and Perm Cell Permeabilization Kit, Thermo Fisher Scientific, Waltham, Massachusetts) then stained for IL-5 (all antibodies from BD Biosciences, Franklin Lakes, New Jersey).

All patients seen in our center in whom the presence of circulating CD3^−^CD4^+^ T cells has been confirmed in association with blood (above 0.5 G/L or 10% WBC) and/or tissue eosinophilia in the absence of an underlying malignant hematological disorder at diagnosis have been included in this retrospective observational study.

Of the 26 patients included in our cohort, 3 were referred to our center and seen punctually for advice and/or treatment (P24-26). The remaining 23 patients are or were seen in our center on a regular basis. Three of these patients (P2, P4, P14) are currently followed elsewhere, but recent updates were obtained through their hematologists. Clinical and laboratory data, as well as treatment history were collected after chart review and compiled in a database without identifiers. For the 3 referred patients, most of the data was obtained through physicians in their home country (The Netherlands for P24 and P25, Denmark for P26). The duration of follow-up was determined as follows: the moment when investigation of HE and associated symptoms (when present) was initiated marks the start date, and June 2019 marks the end date. For patients who have deceased (P1, P10, P25), and those that are either lost to follow up (P24) or for whom we have had no contact for more than 1 year (P2, P7), the end date is date of last contact. Seven patients have been included in previous publications (P1, P2, P3, P4, P5, P10, P24) (4, 7, 11–13).

Approval for conducting this retrospective study was obtained from the Hôpital Erasme's institutional review board. Written informed consent was obtained from living patients and/or legal guardian/next of kin for minors for the publication of any potentially identifiable images or data included in this article.

### Laboratory Assessment on Peripheral Blood and Histopathological Analysis

Results of laboratory analyses were extracted from medical files with the exception of serum CCL17 (thymus and activation-regulated chemokine, or TARC) levels. Serum IgG and IgM immunoglobulins were measured in our hospital's Laboratory of Immunology by nephelometry on a BNII instrument following manufacturer instructions (Siemens Healthcare, Germany), and IgE levels by Fluorimetric Enzyme-Linked Immunoassay. Serum protein electrophoresis was performed at least once in all patients. Pre-treatment values for leukocyte counts and immunoglobulins are those at the time CD3^−^CD4^+^ T cells were first detected, except in patients receiving treatment at that time. For the latter, values are those observed during active untreated disease before detection of abnormal T cells.

Because of the retrospective nature of this study and the long time-span, techniques used for assessment of T cell phenotype and TCR gene rearrangement patterns in whole blood have changed over time. The current flow cytometric protocol for staining and acquisition was introduced in 2004. Together with the nine 5-color antibody combinations used for lymphocyte phenotyping detailed in Supplementary Table 1, acquisition of a large number of cells (roughly 20,000 CD4^+^ lymphocytes), allows optimal detection of CD3^−^CD4^+^ T cells. Flow cytometry instruments and associated data analysis software have changed during this study, consisting of the FACSCanto II from BD Biosciences (BD FACSDiva Software) and more recently the Navios from Beckman Coulter (Kaluza Analysis Software). The majority of TCR gene rearrangement analyses have been performed in whole blood by PCR analysis in accordance with BIOMED-2.

The progression of absolute CD3^−^CD4^+^ T cell counts over time was retrospectively assessed as follows: the baseline count is that at time of first detection, counts at year 1 were collected within a window of 3 months before and after baseline, counts at years 3 and 5 were collected within a window of 6 months before and after the baseline count, and the last count was that at last follow-up beyond 5 years. For patients P2 and P10 who developed lymphoma, the last count is that at time of lymphoma diagnosis. For patient P25 who was treated with ASCT, the last count is that recorded just before this procedure. The TCR gene rearrangement pattern is that observed in whole blood for beta (β) and/or gamma (γ) chain genes when CD3^−^CD4^+^ T cells were first detected, or at the closest time-point available.

Serum was collected for all patients when they first presented in our center and stored at −20° for future determination of serum CCL17/TARC levels using a commercialized ELISA kit (R&D Systems Europe, Abingdon, UK). Pre-treatment serum TARC values were measured in samples collected before initiation of treatment. If no sample was available prior to initiation of therapy, only the peak value measured among subsequent samples is mentioned.

Tissue biopsies performed as routine diagnostic procedures were retrieved from pathology department archives and reviewed by the pathologist contributing to this study (ALT).

### Treatment Response and Disease Course

Responses to individual treatment modalities were analyzed provided the treatment duration and dosing were sufficient, and the quality of data retrieval was such that an accurate assessment could be made retrospectively. The most commonly used oral corticosteroid (OCS) in our cohort was methylprednisolone, and doses were expressed as prednisone (PDN)-equivalent in milligrams (4 mg methylprednisolone is equivalent to 5 mg prednisone). The minimally effective dose (MED), defined as the lowest OCS dose shown to maintain a complete hematological (i.e., normalization of blood eosinophil counts) and clinical remission during the tapering process, was determined as accurately as possible on the basis of clinical records and blood results. For some patients the MED could not be determined for various reasons including dynamic tapering, and variable dosing over time in patients with spontaneous fluctuations of eosinophilia and/or episodic symptoms. Most of the second-line agents were administered as add-on therapy to OCS and/or other CS-sparing agents. Treatment responses were defined as follows: complete response if symptoms were controlled and eosinophil counts normalized (below 0.5 G/L), partial response if symptoms improved but persisted to some extent and/or eosinophil counts remained elevated but dropped by at least 50% compared to pre-treatment levels, no response if neither symptoms nor eosinophil counts were impacted by treatment.

Disease course was studied in patients with at least 1-year follow-up after appearance of disease-related symptoms and HE (or HE alone in subjects with HE_US_), for whom longitudinal data was available in terms of clinical manifestations, biological features, and outcome.

### Statistical Analysis

Statistical analyses were performed using SPSS v26 and graphics were designed with Graphpad Prism v8.2 software. Non-parametric comparisons of group means were made by using the Mann-Whitney *U*-test or the Kruskal-Wallis test when appropriate. Proportions were compared by using the Fisher exact test. A *P*-value less than 0.05 was considered significant.

## Results

### General Presentation of Our Cohort of Patients With CD3^−^CD4^+^ T Cell Associated Hypereosinophilia

Our cohort is composed of 26 patients, among which two (P22, P26) are classified as hypereosinophilia of undetermined significance (HE_US_) because they remain asymptomatic despite persistent (8 and 5 years) and marked HE (peak at 11.15 and 12.4 G/L). The 24 other patients all have symptoms and are classified as L-HES, although two (P6, P21) have never reached blood eosinophil counts defining HE (at least 1.5 G/L) despite numerous CBCs. Blood eosinophilia was >10% at presentation in both, and a pre-treatment skin biopsy showed a dense eosinophilic infiltrate in P2, thereby fulfilling the current diagnostic criteria for HES. One patient (P25) who was referred to our center for expert advice differed from the rest of the cohort in that membrane CD3 expression on her abnormal CD4 T cells was dim but not completely abolished (see Supplementary Figure 1). This abnormal population stained weakly but homogenously for the TCR Vβ4 family.

This condition is equally distributed among males (13) and females (13), and the median age at diagnosis of L-HES is 43.5 years (range 15–78) (Table 1). Four patients experienced their first symptoms before age 20. The median delay between inaugural HES-related symptom(s) and detection of CD3^−^CD4^+^ T cells leading to diagnosis of L-HES is 3.5 years (range 0–18 years). The median duration of follow-up after initiation of investigations for HES in our series is 7.1 years (range 8 months to 31 years), with three patients who have deceased and one subject lost to follow-up.

**Table 1:** Epidemiology and clinical features of patients with a CD3^−^CD4^+^ T-Cell mediated hypereosinophilic condition.

**Pat**.	**Sex**	**Age (years)**			**FU^c^**	**Present sym**	**Clinical manifestations**								**Tx reg**	**Outcome**
		**Sym**	**HE**	**Diag^b^**			**General**	**Skin^d^**	**Angioed^e^**	**Pulm**	**Vascul**	**Rheum**	**Lymph**	**Other**		
P01^a^	M	29	29	30	3.4	Pruritus, dyspnea, NSw	Fev, NSw LOW	Pap		Infiltrasthm	Digit vascul		Clin		2nd line	LymphomaDeceased
P02^a^	F	15	12	21	12.8	Pruritus, eczema	LOW	EczEry			Pulm embol	TenoS	Clin		2nd line	LymphomaASCT, Cured
P03^a^	F	21	22	22	22.3	Pruritus, rash	Fev	Pap, UrtPla	++ diff	Asthm		MyalgTenoS	Clin	Smeg	2nd line	
P04^a^	F	49	47	58	28.4	Pruritus, urticaria		Urt					Isot		No T_x_	Spont clinic remission
P05^a^	M	31	33	43	27.8	Hand angioed	Fev, fat		++ extr			Myoed			2nd line	Cured
P06	M	31	43^f^	43	14.2	Pruritus		Ecz							OCS	
P07	M	24	26	33	18	Facial angioed		Urt, Ecz	+ face				Clin		No T_x_	Spont clinic remission
P08	F	30	27	42	8.8	Facial angioedpruritus			+ face		Rayn	Myalg			OCS	
P09	F	41	48	51	8.8	Bullous lesions	Fat	Bull			Rayn	TenoS	Isot		No T_x_	
P10^a^	F	49	49	50	7	Pruritus, myalgia, abd pain, NSw	Fev, NSw fat	Pap	+++ diff		Rayn	Myositis(**F**-PET)	Isot	hypoNaabd swell	2nd line	LymphomaDeceased
P11	F	40	40	43	7.1	Angieod, myalgia	Fat	Urt, Mac	++ diff		Rayn	Fasc(MRI)			2nd line	
P12	F	36	36	47	8.2	Facial angioed, rash	Fev, fat	Ecz, Mac	+++ extr		Rayn	Fasc(biopsy)			2nd line	
P13	M	57	57	58	5.2	Pruritus, rash		Pap		Infiltr			Isot	Sinuspolyp	2nd line	
P14	M	52	50	53	5.1	pruritus, angioed	Fat		++ extr			Myoed	Isot	Sub-cut nod	OCS	
P15	F	45	43	45	5.3	Facial angioed	Fat	Mac	+ face, extr						No T_x_	
P16	F	32	38	42	4.3	Facial angioed		Ecz, Urt	+ face						No T_x_	
P17	M	35	35	42	2.5	Cholestasis		Ecz					Isot	cholang	No T_x_	
P18	F	65	65	66	3	Pruritus, rash		Ery					Isot		2nd line	
P19	M	65	64	68	4.8	Pruritus, urticaria		Urt						EoESinus	OCS	
P20	M	74	77	78	2.5	Pruritus		Ecz						Sinus	No T_x_	
P21	M	63	63^f^	65	3.1	Pruritus		Ery					Clin		OCS	
P22	M	NA	37	44^g^	7.4	NA									NA	
P23	M	16	15	16	0.7	Hand angioed			+ extr				Isot	Smeg	2nd line	
P24 ^a^	F	16	19	34	22.3	Arthro-myalgia, rash	Fev, fat	Mac	++ diff			Myoedarthral			2nd line	Lost to FU
P25	F	13^h^	7	15	10.2	Pruritus, rash	Fat	Pap	+++ diff	Asthm	Rayn dissec	Fasc(biopsy)	Clin	abd swellSmeg, (uveit)	2nd line	ASCTDeceased
P26	M	NA	45	48^g^	5.4	NA									NA	

a *Cases previously reported in refs 4 (P1), 7 (P1-P4), 11(P5), 12 (P10), and 13 (P24)*.

b *Age at diagnosis of L-HES*.

c *Duration (years) of follow-up after initial work-up for combined hypereosinophilia and symptoms (when present)*.

d *Skin lesions include bull, bullous; ecz, eczema; ery, erythroderma; mac, macules; pap, papules; pla, plaques; urt, urticaria*.

e *Angioedema was mild (+), moderate (++), or severe (+++) and involved the face and/or extremities (extr) eventually with an extension to the abdomen (diffuse)*.

f *Eosinophilia < 1.5 G/L, but > 10% of WBC*.

g *Age at discovery of CD3^−^CD4^+^ T cells*.

H *uveitis at age of 7 when HE first discovered; unclear whether this was eosinophil-mediated as uveitis never recurred thereafter*.

*abd, abdominal; angiod, angioedema; arthral, arthralgia; ASCT, allogeneic stem cell transplantation; asthm, asthma; cholang, eosinophilic cholangitis; clin, clinically palpable lymphadenopathy; diagn, diagnosis; dissec, dissection of vertebral artery; EoE eosinophilic esophagitis; F-PET, fluoro-deoxyglucose positon emission tomography; fasc, fasciitis; fat, fatigue; fev, fever; FU, follow-up; HE, hypereosinophilia; hypoNa, hyponatremia; infiltr, infiltrates; isot, isotopic (hypermetabolic lymph nodes seen on FDG-PET); LOW, Loss of weight; lymph, lymphadenopathy; MRI, magnetic resonance imaging; myalg, myalgia; myoed, myoedema (muscle swelling and induration); NA, not applicable; nod, nodules; NSw, night sweats; Pat, patient number; Present sym, presenting symptoms; OCS oral corticosteroid monotherapy; pulm, pulmonary; Rayn, Raynaud's; rheum, rheumatological; Smeg splenomegaly; tenos, tenosynovitis; Tx (reg), treatment regimen; uveit, uveitis; vascul, vascular*.

### Clinical Manifestations of Disease

**Figure 1:**
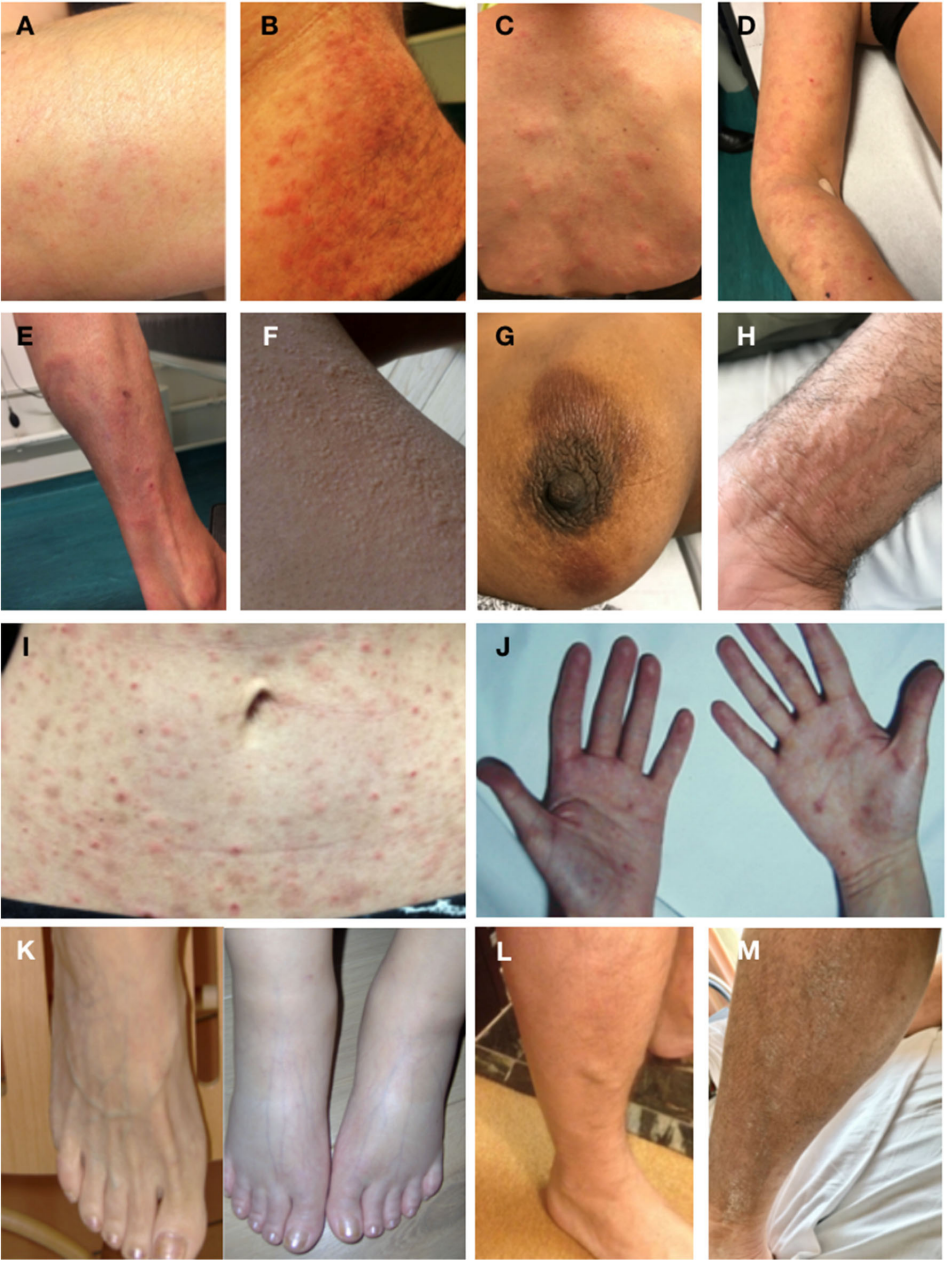
Spectrum of cutaneous and soft tissue manifestations observed in patients with CD3^−^CD4^+^ T cell associated L-HES. **(A)** P12, rash on arm lasting less than 1 day. **(B)** P13, confluent localized papules on abdomen lasting several days. **(C,D)** P3, disseminated migratory papules on back and arm. **(E)** P3, large fixed cutaneous plaque lesion on lower leg lasting months. **(F)** P16, urticaria on thigh, lasting minutes. **(G)** P16, two fixed pigmented plaques around nipple. **(H)** P21, fixed plaque. **(I)** P25, disseminated migratory papules on abdomen. **(J)** P2, scaly eczema hands lasting years. **(K)** P10, feet between and during flares of episodic angioedema. **(L)** P14, diffuse angioedema of lower leg with more localized lumps lasting several hours. **(M)** P21, diffusely lichenified hyper-pigmented skin.

**Figure 2:**
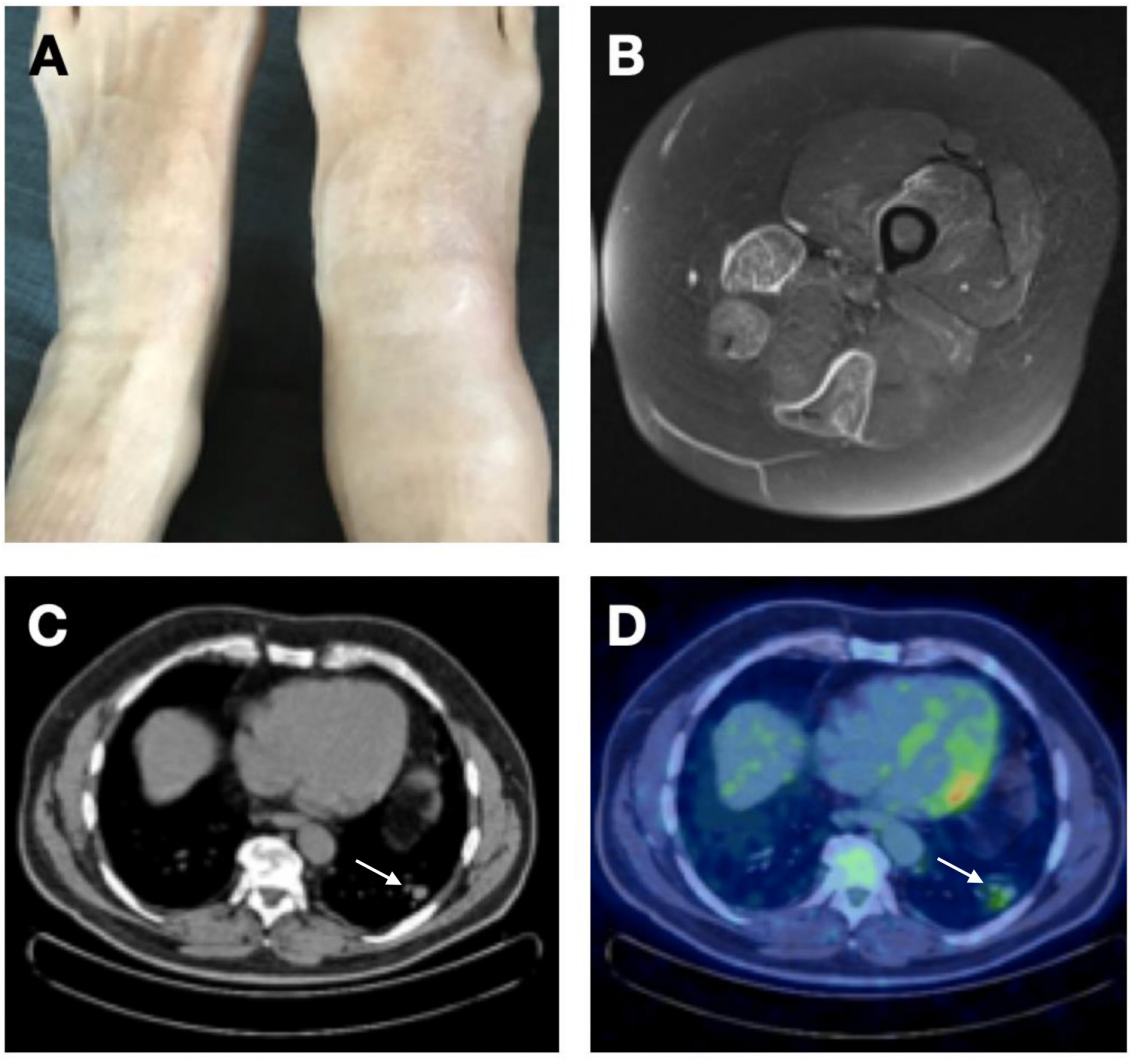
Non-cutaneous complications of CD3^−^CD4^+^ T cell associated L-HES. **(A)** P3, arthritis and synovitis right ankle. **(B)** P11, fasciitis—MRI showing gadolinium enhancement of the fascia and underlying sartorius and gracilis muscles of right leg. **(C,D)** P13, pulmonary involvement—Thoracic CT **(C)** scan showing nodule in left lower lobe (see white arrow), with mild to moderate hypermetabolic activity shown by FDG-PET scan **(D)**.

Disease manifestations in the 24 symptomatic patients are summarized in Table 1 and illustrated in Figures 1, 2. The most common inaugural symptom is pruritus (*n* = 14) associated with skin lesions (*n* = 7), angioedema (*n* = 2), or general symptoms (*n* = 2), and in some cases in the absence of other manifestations (*n* = 3). The next most common symptoms at presentation are cutaneous lesions (*n* = 10) and angioedema (*n* = 9).

**Figure 3:**
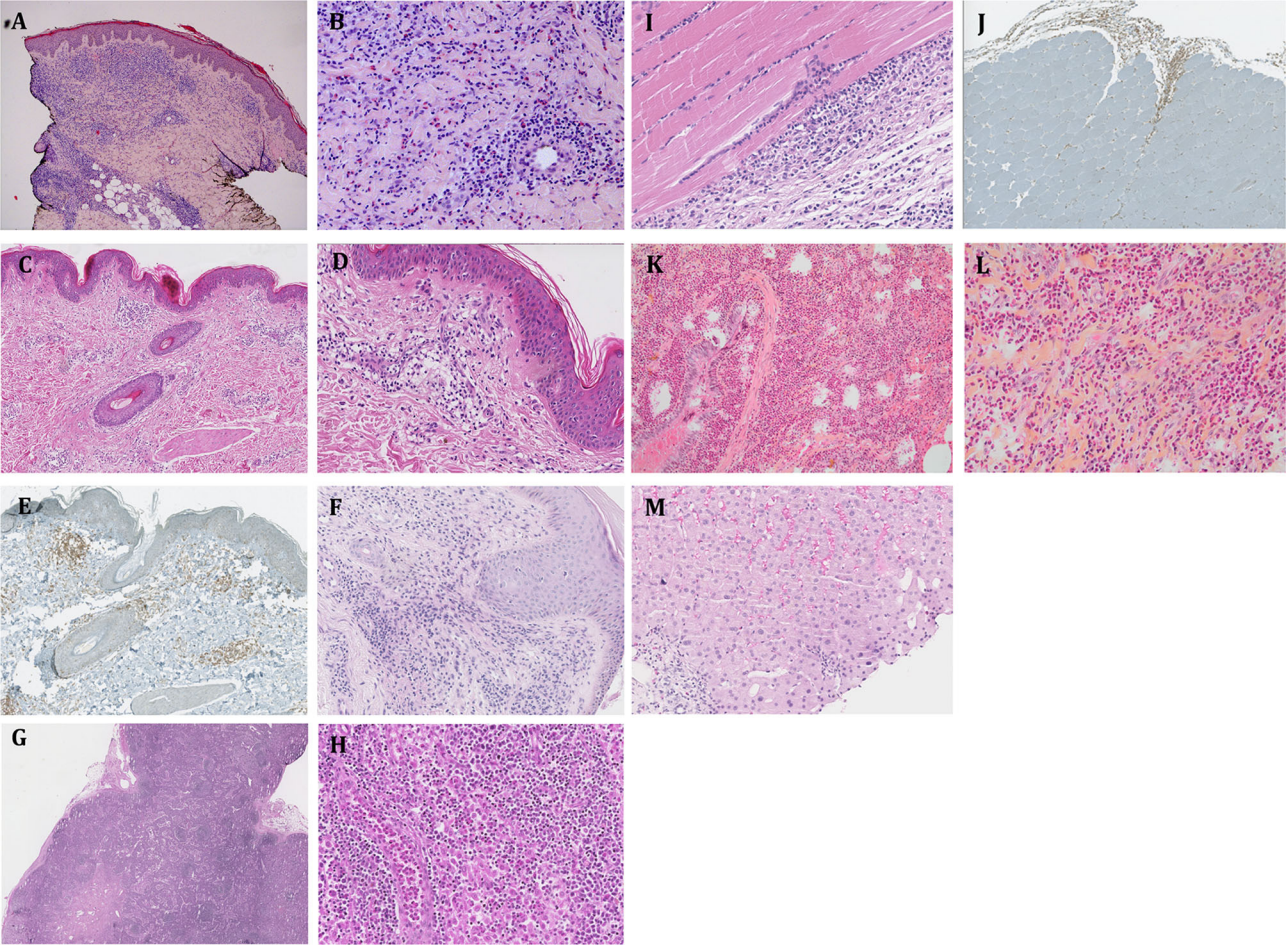
Biopsy findings in patients with CD3^−^CD4^+^ T cell-associated L-HES. **(A,B)** P3, skin biopsy (thigh) at site of persistent papular eruption despite OCS therapy: hematoxylin-eosin stain showing dense lymphocytic and eosinophilic infiltrate of upper and lower dermis (blood eosinophils elevated); 25-fold **(A)** and 200-fold **(B)** magnification. **(C–E)** P25, skin biopsy (thigh) at site of papular eruption before initiation of OCS therapy: hematoxylin-eosin stain showing moderate lymphocytic infiltrate of upper and lower dermis in the absence of eosinophils (although marked blood hypereosinophilia present at the time); combined immuno-staining for CD4 (brown) and CD8 (red) **(E)** showing that the lymphocytes are CD4 cells; 40-fold **(C,E)** and 100-fold **(D)** magnification. **(F)** P6, skin biopsy (popliteal region) at site of persistent fixed eczematous plaque despite ongoing OCS treatment: hematoxylin-eosin stain showing a dense lymphocytic infiltrate of the upper and lower dermis (blood eosinophils normal); 100-fold magnification. **(G, H)** P3, biopsy of enlarged lymph node (inguinal region): hematoxylin-eosin stain showing a dense inflammatory infiltrate and marked dilatation of sinusoid vessels containing numerous eosinophils; 25-fold **(G)** and 200-fold **(H)** magnification **(I,J)** P25, fascia-muscle biopsy (thigh): hematoxylin-eosin stain showing a dense inflammatory infiltrate of the fascia and perimysium, composed of lymphocytes, eosinophils, and plasma cells; combined immuno-staining for CD4 (brown) and CD8 (red) **(J)** showing that the lymphocytes are CD4 cells; 200-fold **(I)** and 40-fold **(J)** magnification. **(K,L)** P13, lung biopsy: hematoxylin-eosin-safran stain showing a dense intra-alveolar eosinophilic infiltrate; 100-fold **(K)** and 200-fold **(L)** magnification. **(M)** P17, liver biopsy: hematoxylin-eosin stain showing an intrasinusoidal and portal-space inflammatory infiltrate composed of lymphocytes and eosinophils; 200-fold magnification.

As disease develops, one or several tissues may be affected and symptoms evolve accordingly. Skin lesions are the most prevalent disease manifestation, observed in 20/24 (83%) patients. The nature of cutaneous symptoms varies considerably, ranging from episodes of mild transient rash to generalized erythroderma. Some patients have urticaria, others extremely pruritic disseminated migratory erythematous papules (centimetric) that last several days/weeks with spontaneous (generally brief) improvement, and yet others present with localized plaques resembling eczema (lichenified erythematous skin, some with increased pigmentation) that are larger (decimetric) and chronic (lasting months or years). A single patient presented with several consecutive bullous lesions that have not recurred despite absence of treatment for 8 years. Skin biopsies, performed in 13 subjects, generally show mixed perivascular, periadnexal dermal inflammation involving eosinophils and mononuclear cells (Figure 3). The latter are predominantly CD4^+^ T cells, and TCR gene analyses (when performed) confirm the presence of the same clonal rearrangement as observed in peripheral blood (when present, see below).

A significant proportion of patients with L-HES have episodic angioedema (54%). The swelling may involve the face (*n* = 3), extremities (distal to elbows and knees, *n* = 4), or both (*n* = 1), or it may be diffuse, also involving proximal extremities and the abdomen with significant weight gain (*n* = 5). One patient with moderate swelling of his extremities also develops more localized lumps under the skin that last several hours or days (Figure 1L). Patients with marked angioedema may also present with low-grade fever, a fleeting rash and/or urticaria during flares.

Half of the patients in our cohort had lymphadenopathy. Although peripheral lymph nodes were found on clinical examination only in 6 subjects, fluorodeoxyglucose positron-emission-tomography (FDG-PET) revealed the presence of small hypermetabolic lymph nodes in 7 additional cases. When present, clinically detectable lymphadenopathy is mild. Biopsy shows diffuse intrasinusoidal infiltration by eosinophils (Figure 3).

Episodic muscle pain and induration involving arms and/or legs was present in 7 patients, with confirmation of fasciitis by MRI (*n* = 1, Figure 2), muscle biopsy (*n* = 2, Figure 3), or PET-FDG (*n* = 1) in some cases. Serum creatine kinase and troponin levels were normal, and serum aldolase (not routinely available) was slightly elevated in 2 of the 3 patients tested in this series. Recurrent (teno)synovitis occurred in three subjects (Figure 2), with the presence of CD3^−^CD4^+^ T cells in synovial fluid in the two patients who were investigated.

None of the patients in our cohort had anomalies suggestive of eosinophil-mediated cardiac involvement, as assessed by echocardiograms and/or cardiac MRI (performed in all but 3 subjects, P17, P21, P22, whose serum troponin levels were normal). Vascular manifestations were observed however, consisting most frequently in Raynaud's phenomenon (*n* = 6). Digital thrombotic vasculitis occurred in one patient, dissection of an aneurysm of the right posterior inferior cerebellar artery in another, and a third patient experienced otherwise unexplained pulmonary embolism. Four patients presented with respiratory symptoms early in disease course: two with isolated asthma, one with eosinophilic pulmonary infiltrates (Figures 2, 3), and one with both asthma and infiltrates. The youngest patient in our series presented at 7 years of age with uveitis; at the time she had increased blood eosinophilia (1.69 G/L). It remains unclear however whether this was already a manifestation of L-HES, as she remained asymptomatic after that single episode for 6 years and then developed cutaneous lesions and angioedema, both symptoms that persisted throughout disease course in the absence of uveitis.

### Hematological and Immunological Findings

Pre-treatment blood leukocyte counts and serum immunoglobulin levels are shown in Table 2. The median lymphocyte count was 2.09 G/L, with only two patients presenting lymphocytosis, while two actually had persistently reduced total lymphocyte counts. The median eosinophil count was 3.81 G/L (range 0.6–53.03 G/L), reaching a median peak level of 8.45 G/L (range 0.77–53.03 G/L) during follow-up, with 12/26 (46%) patients exceeding the threshold of 10 G/L. Serum IgE levels were increased in just over half of our patients (14/25 subjects, 56%), with a median level of 231 KU/L (range <2 to >5,000). Polyclonal hypergammaglobulinemia was observed in 13/26 (50%) of cases, with increased serum IgG alone in 5, IgM alone in 4, and both IgG and IgM in 4. Other biomarkers considered helpful for HES classification (namely serum tryptase and TARC) are shown in Supplementary Table 2. Pre-treatment routine chemistry results were unremarkable in our cohort with the exception of mild elevations of serum LDH levels in 16/24 (67%) and CRP levels in 2/23 patients for whom values were available (not shown).

**Table 2:** Biological features of patients with CD3^−^CD4^+^ T-cell associated hypereosinophilia.

**Pat**	**Routine biology**					**Max HE**	**T cell investigations in blood**		
	**Lympho G/L**	**Eosino G/L**	**IgE KU/L**	**IgG g/L**	**IgM g/L**	**G/L**	**CD3^−^CD4^+^ % lympho**	**CD3^−^CD4^+^ G/L**	**TCR rearr^b^ (β and/or γ)**
P01	1.47	3.76	1061	15.5	72	12.35	43	0.63	+
P02	4.67	5.54	340	7	3.1	17.10	71	3.00	+^c^
P03	2.86	11.61	10360	22.26	22.6	11.61	60	1.72	+
P04	2.24	2.97	478	19.2	2.36	5.50	10	0.22	–
P05	2.12^a^	19.28^a^	15^a^	12.7	7.74^a^	19.28	30^a^	1.73^a^	+^a, c^
P06	1.37	0.77	7849	10.3	1.23	0.77	8.4	0.12	+
P07	1.98	0.71	13	9.48	1.87	9.60	0.5	0.01	–
P08	1.78	7.35	2896	14.8	1.66	13.80	1.9	0.03	(+)
P09	1.97	4.47	45^a^	14	1.24	5.00	7	0.14	(+)
P10	1.70	2.14	838^a^	55.3^a^	11.5^a^	15.00	2	0.03	–
P11	1.43	2.00	57^a^	17.2	6.39	4.20	0.72	0.01	(+)
P12	2.43	6.09	225	16.7	2.24	14.04	23	0.56	+
P13	1.70	2.40	>5000	11.9	0.78	4.16	29^a^	1.15^a^	+^a^
P14	2.00	3.48	79^a^	17.4	1.18	4.10	71	1.42	+
P15	2.22	2.40	15	14.9	0.86	6.20	1.18	0.03	–
P16	0.78	0.60	326	13.4	1.12	1.56	7.37	0.06	+
P17	3.13	3.00	>5000	33.6	1.11	5.50	0.46	0.01	–
P18	1.24	1.17	66^a^	13	0.97	4.60	13	0.16	(+)^a, c^
P19	1.94	5.84	19	11.3	1.31	7.29	0.15^a^	0.002^a^	–^a^
P20	2.45	6.36	<2	14.3	2.17	22.40	2.7	0.07	+
P21	1.56	0.90	12	18.2	1.38	0.90	22	0.34	+
P22	3.86	11.15	869	12.5	0.9	11.15	0.7	0.03	–
P23	3.54	53.03	nd	7.6	10.76	53.03	0.86^a^	0.03^a^	–^a^
P24	3.17	4.20	22	12.2	2.42	40.00	0.46	0.02	–
P25	4.77	3.87	461	14.7	3.4	6.27	57	2.71	+
P26	2.97	10.60	231	10.5	1.27	12.40	1	0.03	–^c^

a *Maintenance treatment ongoing at time assessment was performed*.

b *Results for TCR gene rearrangement patterns are recorded as follows: + clonal pattern, (+) clonal peak on a polyclonal background, - polyclonal*.

c *Not assessed at time of discovery of CD3^−^CD4^+^ T cells; result shown is the first assessment thereafter*.

*Normal values: lymphocytes 1.340–3.950 G/L; eosinophils 0.1–0.5 G/L; IgE < 120 KU/L; IgG 6–15 g/L; IgM 0.4–2.5 g/L*.

*HE, hypereosinophilia, nd, not done, Tx, treatment, rear, rearrangement*.

**Figure 4:**
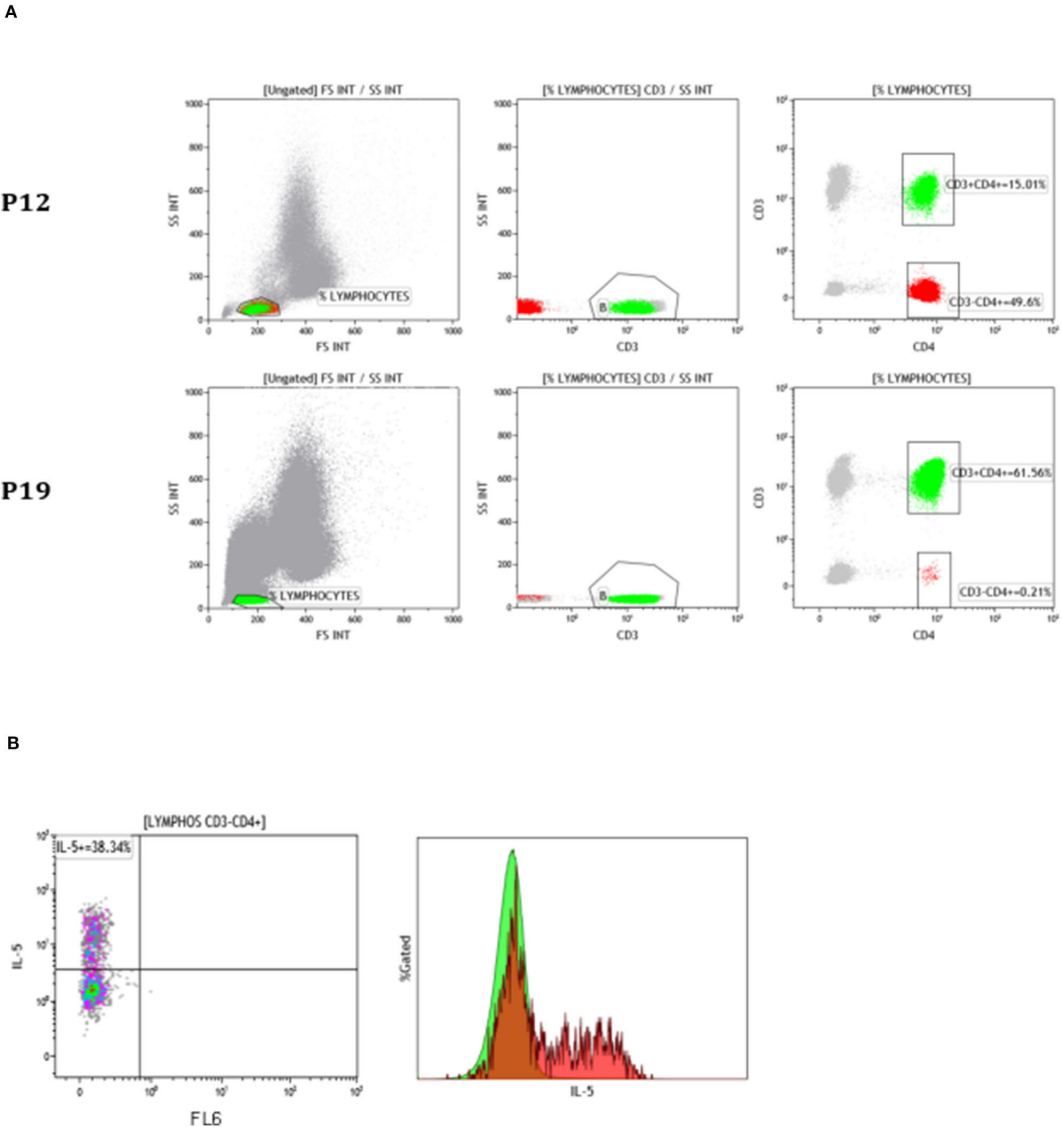
T cell phenotyping by flow cytometry in 2 representative patients with a CD3^−^CD4^+^ T cell subset. **(A)** T cell phenotyping by flow cytometry showing a large (P12, top) and a small (P19, bottom) CD3-CD4+ T cell subset. The plots show the gating strategy from left to right: gate on lymphocytes on the basis of forward- and side-scatter, after selecting CD45hi cells (not shown) CD3 expression is plotted against side-scatter and CD3+ cells (green) are differentiated from CD3- cells (red), the proportions of CD3^+^CD4^+^ and CD3^−^CD4^+^ T cells among CD45hi cells are shown on the right. **(B)** Intracytoplasmic IL-5 expression was assessed in CD4 T cells from patient P19. The histogram overlay shows IL-5 expression in CD3^+^CD4^+^ T cells (green) and in CD3^−^CD4^+^ cells (red).

The T cell investigations leading to diagnosis of L-HES include T cell phenotyping and TCR gene rearrangement analysis (Table 2). With the exception of 4 patients (P5, P13, P19, P23), the CD3^−^CD4^+^ T cell subset was detected before initiation of therapy. The initial median percentage of CD3^−^CD4^+^ T cells among total lymphocytes was 7.2% (range 0.15–71%), representing a median absolute count of 0.091 G/L (range 0.002–3). The abnormal T cells represented 2% or less of total lymphocytes in 11 (42%) patients in our cohort (Figure 4). Assessment of T cell clonality by TCR gene rearrangement analysis on whole blood was available for 22 subjects at the time CD3^−^CD4^+^ T cells were discovered, and the 4 remaining patients were assessed at later time-points. A clear-cut clonal pattern was observed in 12 (46%), a small clonal peak on a polyclonal background (result considered equivocal) in 4, and the 10 remaining subjects had a normal polyclonal pattern. TCR gene assessments were repeated at least once in 21 patients (Supplementary Table 2), showing an identical rearrangement pattern over time in those with demonstrated clonality.

Karyotypes were available for 16 subjects and found to be abnormal in 2 (P2, P3). Both subjects had partial deletions on chromosome 6q. Patient P2 also had a partial deletion on 10p. Bone marrow sampling was performed in 19 patients (15 biopsies and 4 bone marrow aspirations) showing increased eosinophilia in all cases and presence of CD3^−^CD4^+^ cells when assessed (6 cases). No other anomalies were observed, with the following exceptions: P9 had a slight excess of morphologically normal mast cells, and P18 had hyperplasia of all 3 lineages (in the presence of a V614F JAK2 mutation).

### Therapeutic Management

**Figure 5:**
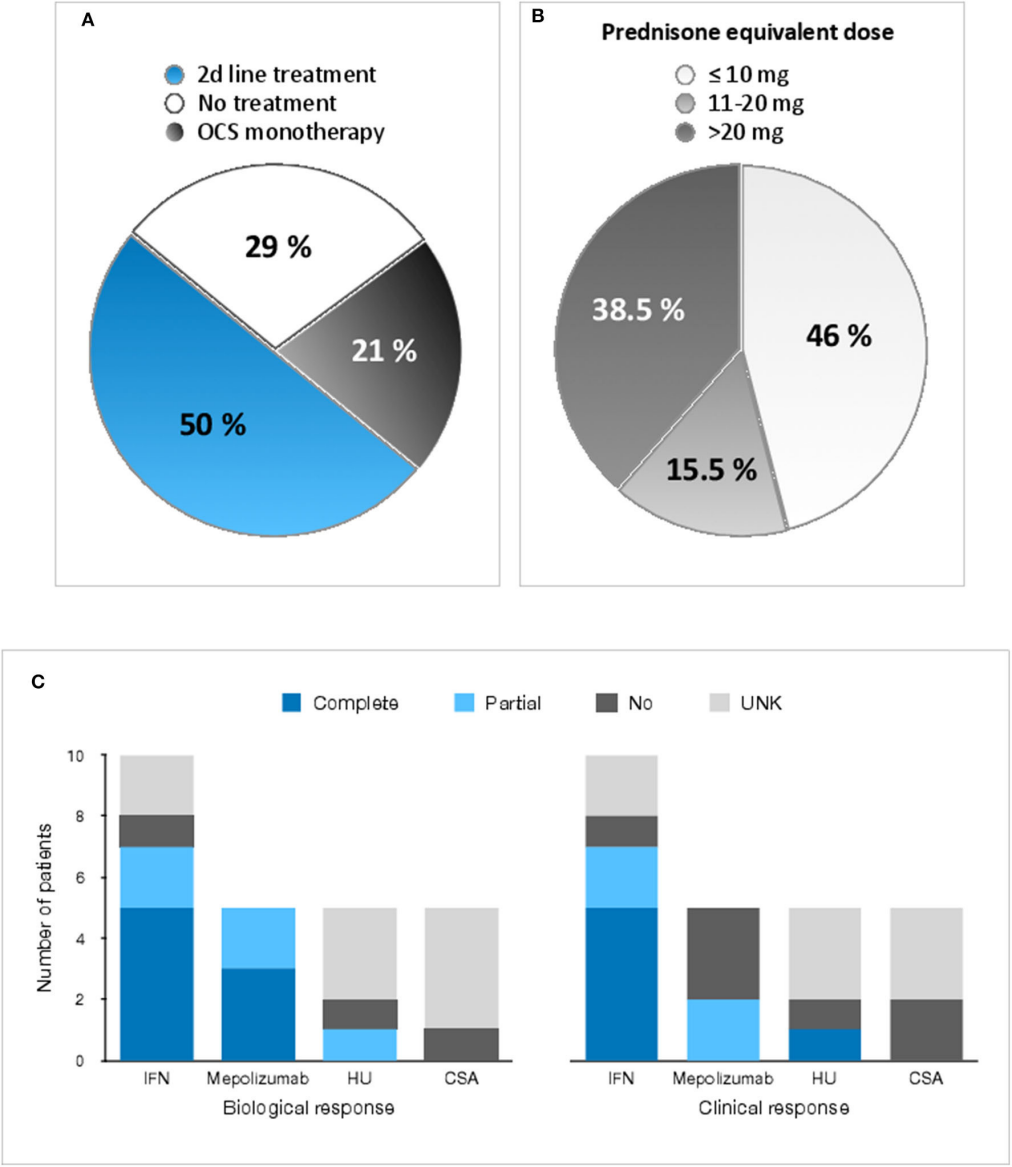
Treatment regimens and responses in patients with CD3^−^CD4^+^ T cell associated L-HES. **(A)** Proportions of patients who required no maintenance treatment (white), prolonged OCS monotherapy (gray), or addition of a second-line agent (blue) to control disease. **(B)** shows the minimally effective dose of oral CS as monotherapy for a complete response early in disease course in the 13 patients for whom this dose could be determined accurately (one patient was already treated with HU when OCS were initiated, and OCS could not be increased to a dose such that a complete response occurred in two others due to marked intolerance). Proportions of patients who experienced a complete response with a PDN-equivalent dose at 10 mg or less (light gray), 11–20 mg (medium gray), and above 20 mg (dark gray) daily are depicted. **(C)** shows responses to the most commonly administered second-line agents, separating biological (left) from clinical (right) responses. A partial response is defined as a reduction of eosinophil counts by at least 50% with treatment (biological) and persistent but improved symptoms (clinical). A complete response is defined as normalization of blood eosinophil counts (biological) and resolution of HES-related symptoms (clinical). CSA cyclosporin A, HU hydroxyurea, IFN interferon, UNK unknown. See Supplementary Table 5 for individual data.

Symptomatic patients (*n* = 24) were classified on the basis of therapeutic regimens as follows: (1) no maintenance therapy, (2) prolonged OCS monotherapy, and (3) administration of second-line therapy at some point during disease course. Patient distribution among these 3 categories is shown in Table 1 and Figure 5A.

Seven patients (29%) have not received maintenance therapy. These patients either have mild and infrequent episodic cutaneous and/or sub-cutaneous symptoms, or they have plaques resembling eczema on a limited surface of their skin. The former occasionally resort to short-term OCS use during flares, and the latter to topical CS.

For 5 patients (21%), maintenance therapy to this day has consisted in OCS alone. The reason for treatment was chronic dermatitis in 3 (P6, P19, P21), and persistent angioedema in 2 (P8, P14). The median duration of OCS monotherapy was 55 months (range 14–174 months).

Finally, 12 patients (50%) have required second-line therapy. With the exception of P18 who was treated with hydroxyurea because investigations revealed thrombocytosis with a JAK2 V614F point mutation, second-line agents were initiated because of severity of L-HES disease manifestations and/or requirement for high-dose maintenance OCS therapy. The most commonly used second-line agents consist in interferon-alpha (IFN-α) (*n* = 10), hydroxyurea (*n* = 5), mepolizumab (*n* = 5) and cyclosporine A (*n* = 5), followed by IFN-γ (*n* = 2), cyclophosphamide (*n* = 2), imatinib mesylate (*n* = 2), methotrexate (*n* = 2). Various other medications administered to one patient each are enumerated in Supplementary Table 3. A mean of 3.4 different agents were administered per patient. At time of initiation of second-line agents, all patients except three (P2, P11, P18) were receiving maintenance OCS therapy.

Clinical and biological features at presentation were compared among treatment groups to determine if specific presentations were associated with subsequent needs in terms of maintenance therapy. Neither eosinophil and absolute CD3^−^CD4^+^ T cell counts, nor serum TARC levels at presentation differed significantly between treatment groups. In contrast, the pre-treatment serum IgM level was significantly higher in patients requiring second-line treatment (GM serum IgM 1.44 g/L, 1.34, and 6.11 in untreated, OCS monotherapy, and second-line treatment groups respectively, *p*-value 0.005). Given the small sample size, no statistically significant associations between specific disease manifestations and treatment allocation were identified, although all 4 subjects with lung involvement did receive second-line therapy, as did all 3 patients who ultimately developed T cell lymphoma.

### Treatment Responses

Treatment responses were evaluated retrospectively in the patients for whom sufficiently detailed data on eosinophil counts and symptoms before and after initiation of therapy was available. Patient P24 was referred from abroad for anti-IL-5 treatment and seen in our center only twice, so her response to only that specific agent was analyzed.

In all, 16 patients were administered OCS for at least 6 consecutive months with a median initial PDN-equivalent dose of 35 mg (range 10–80) (Supplementary Table 4). None of the patients were OCS-resistant. For the 13 patients in whom OCS were continued long-term, the minimally effective maintenance dose (MED) for a complete initial response was distributed as follows: 5 required more than 20 mg PDN-equivalent daily, 2 required 11–20 mg, and 6 required 10 mg or less (Figure 5B). Over time, dosing requirements changed in some, with four patients whose dose had to be increased to maintain disease control (P3, P5, P12, P19), whereas the dose could eventually be decreased in two subjects (P6, P13) (see details in Supplementary Table 4). All patients with a MED initially above 10 mg PDN daily have received second line agents.

The most commonly administered second-line therapies are IFN-α, HU, mepolizumab, and cyclosporin A. Treatment responses are shown in Figure 5C and detailed in Supplementary Table 5. Of the 10 patients treated with sub-cutaneous injections of IFN-α, 3 received the pegylated form (Pegasys®, weekly injections) and 7 received recombinant IFN-α-2b (Intron-A®, several injections a week). The 8 subjects in whom the response to treatment could be analyzed reliably experienced variable degrees of improvement. Three patients had a complete response, ultimately leading to disease cure in one case (P5) with disappearance of CD3^−^CD4^+^ cells 27 months after introduction of IFN-α. One of the two other complete responders (P23) had to stop treatment after 2 months due to severe neutropenia, while the third (P3) is still receiving pegylated IFN-α 4 months after initiation with no apparent side effects so far. Although partial responses in the five other patients were accompanied by a reduction in eosinophil counts and enabled OCS-sparing, treatment had to be interrupted in all cases, due to (secondary) drug resistance and/or poor tolerance.

Of the 5 patients receiving HU, treatment response could be analyzed in only two subjects who received it as add-on therapy for 3 and 16 months at appropriate dosing. This agent was interrupted in both, either because it was completely inefficacious (P1) or because the marginal benefit of a partial response was out-weighed by poor tolerance (P12). In P18, low-dose HU alone, administered long-term after a 6-month course of OCS for erythroderma, has been associated with a complete remission lasting more than 2 years, although a small contingent of CD3^−^CD4^+^ cells is still detectable. As HU has been pursued to control V614F JAK2-associated thrombocytemia, it has not been possible to establish its contribution to maintenance of HES remission.

Mepolizumab was obtained for 5 subjects in the setting of a compassionate use program (NCT002446686) and administered at high doses (700–750 mg, monthly intravenous infusions) alone (P11) or in association with OCS. A clear-cut hematological response was observed in 4/5 cases with eosinophilia dropping to or below 0.5 G/L, although this response was short-lived in P3 with recurrence of eosinophilia above 1 G/L despite continuous monthly infusions. The clinical response was overall disappointing. Although 2 patients experienced partial improvement of angioedema, symptoms of associated muscle involvement were unchanged (P10, P11). Two patients actually experienced progressive worsening of cutaneous manifestations with persistent tissue eosinophilia (P3, P12).

Other second-line agents interrupted after short periods (less than 6 months) due to lack of efficacy include cyclosporine A, methotrexate, IFN-γ, cyclophosphamide, mycophenolate mofetil, imatinib mesylate, tacrolimus, and dupilumab. Patients P2 and P25 had progressive treatment-refractory disease that was judged severe enough to warrant more aggressive therapy despite negative investigations for T cell lymphoma. Four years after diagnosis of L-HES, P2 developed cervical lymphadenopathy and parotid gland nodules, while eosinophil counts increased above 10 G/L and eczema worsened. She received 6 monthly cycles of fludarabine, which resulted in normalization of eosinophil counts and disappearance of clinical manifestations that lasted only 6 months after the last dose. Allogeneic stem cell transplantation was performed in P25 8 years after presentation, resulting in infectious complications and death 2 months after the procedure.

### Clinical Disease Course and Patient Outcome

Patterns of disease course were analyzed over time and are detailed in Supplementary Table 6. Patient P24 was not included in this analysis because data retrieved from abroad was insufficient.

Two patients (P22, P26), classified as HE_US_, remain symptom-free 8 and 5 years, respectively after discovery of marked HE. In 6 additional patients who were diagnosed with HES at the time they consulted for symptoms (angioedema in 4 cases), retrieval of blood tests performed previously revealed that HE was present more than a year prior to clinical manifestations.

At time of inaugural HES symptoms, patients presented variable clinical patterns. Seven had highly symptomatic disease that progressed rapidly (within weeks or months) after initial clinical manifestations and required prompt initiation of systemic therapy (P1, P3, P10, P13, P14, P18, P25). Three of these patients (P3, P14, P18) experienced clinical improvement immediately after introduction of OCS treatment, while the others required second-line therapy shortly thereafter. In contrast, 7 other patients presented with extremely mild cutaneous manifestations that did not warrant maintenance therapy. Subsequently, they either experienced spontaneous and prolonged resolution of symptoms (P4, P7, P9) and eosinophilia (P4, P7) after several years despite persistence of a small contingent of CD3^−^CD4^+^ T cells, or relatively stable disease that has not yet required initiation of treatment (P15, P16, P17, P20). Yet others presented with cutaneous and/or soft tissue manifestations of moderate severity that justified initiation of maintenance OCS, leading to regression of disease manifestations with a low dosing regimen (P6, P8, P21), or followed by progressive worsening over several years eventually requiring therapeutic escalation (P2, P5, P11, P12, P19).

Three subjects developed T cell lymphoma several years after initial presentation. A diagnosis of anaplastic null-cell lymphoma (spleen and bone marrow) was made 4 years after initial symptoms when patient P1 developed fever, weight loss, polyadenopathy, and organomegaly. Peripheral T cell lymphoma not otherwise specified was observed in a cervical lymph node biopsy in patient P2 15 years after her first symptoms, and 6 years after detection of CD3^−^CD4^+^ T cells. Patient P10 developed angioimmunoblastic T cell lymphoma 6 years after initial symptoms, presenting as a single enlarged cervical lymph node. Only patient P2 was cured of disease after treatment with ASCT. Both P1 and P10 died of infectious complications of chemotherapy before a response was observed.

In addition to lymphoma, notable clinical outcomes in our cohort include HES resolution with IFN-α in one subject (P5), and one treatment-related death in a patient who underwent ASCT for extremely symptomatic high-dose-OCS-dependent non-malignant disease and succumbed to infectious complications (P25).

### Longitudinal CD3^−^CD4^+^ T Cell Counts

**Figure 6:**
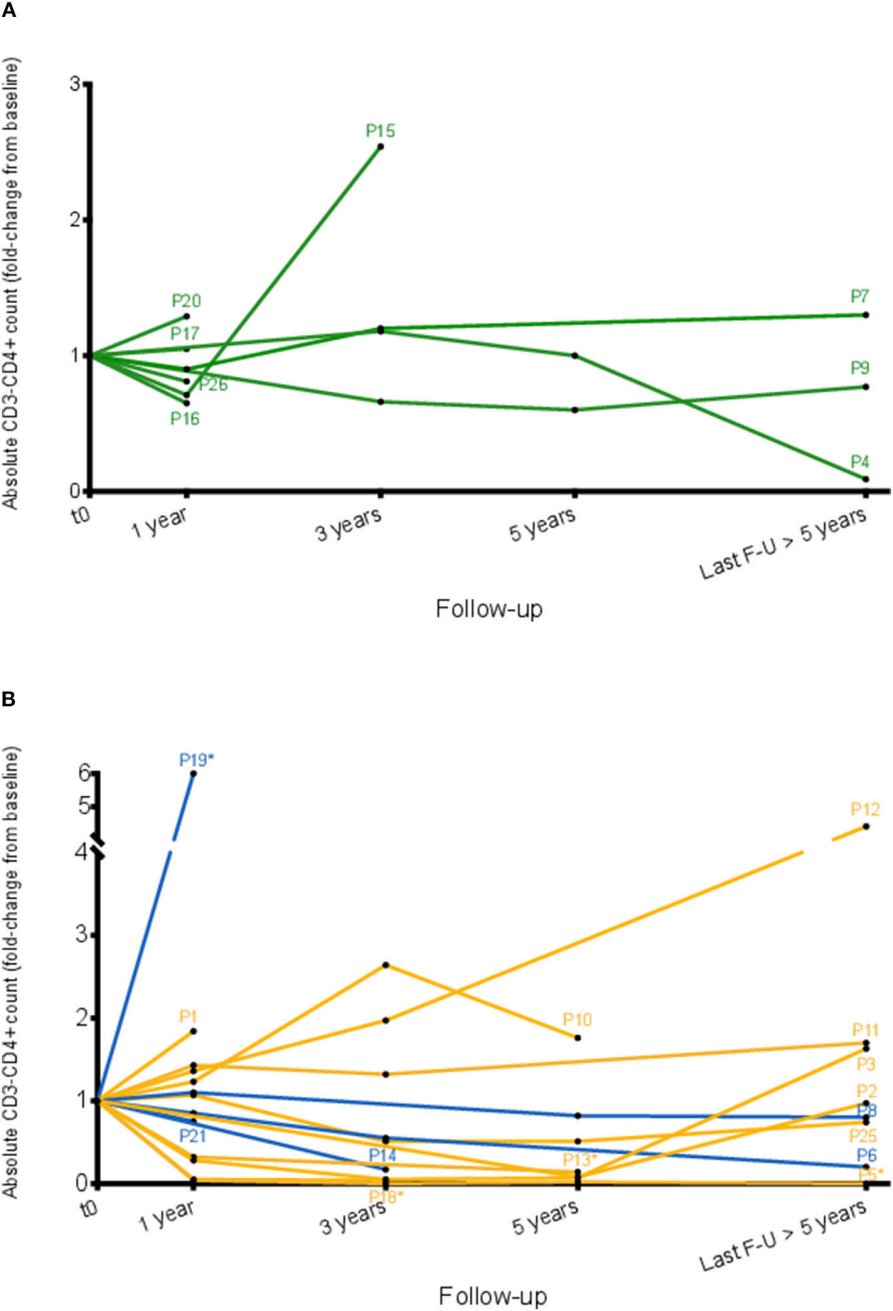
Evolution of absolute CD3^−^CD4^+^ T cell counts over time. Evolution of absolute blood CD3^−^CD4^+^ T cell counts over time is shown for all patients except P22 and P23 for whom duration of follow-up is less than a year, and P24 who is followed in another center. Absolute CD3^−^CD4^+^ T cell counts are normalized relative to baseline counts for each patient (i.e., those at initial detection of these cells), and are shown separately for untreated subjects **(A)** and subjects receiving treatment **(B)** either with oral CS alone (blue lines) or second-line therapy (yellow lines). For the three patients who developed lymphoma (P1, P2, P10) the count at last follow-up is that at the time lymphoma was diagnosed. For the other patients, the most recent CD3^−^CD4^+^ cell count and percentage, as well as the interval since first detection, are shown in Supplementary Table 7. Patients who were treated at baseline are indicated with an asterisk.

Abnormal T cell counts were analyzed over time in patients for whom serial counts were available (*n* = 23), and their evolution is shown in Figure 6 and Supplementary Table 7. Overall, 12/23 patients had relatively stable counts over a median timespan of 51 months (range 10 to 135). Six subjects experienced an at least 1.5-fold increase in CD3^−^CD4^+^ cell counts compared to baseline values, and 5 experienced a decrease to below half of baseline counts, with complete disappearance of these cells in P5.

Disease activity was analyzed with regard to changes in CD3^−^CD4^+^ T cell counts. Among the 6 patients whose CD3^−^CD4^+^ cell counts increased over time, 4 experienced clinical worsening in parallel and required therapeutic escalation. As for the patients whose CD3^−^CD4^+^ counts have dropped, this has paralleled clinical and hematological improvement in all cases, either in response to maintenance treatment (P5, P6, P13, P18) or spontaneously (P4).

The impact of treatment on CD3^−^CD4^+^ cells was studied according to different regimens. Among the 10 patients for whom detailed data on serial CD3^−^CD4^+^ counts and OCS dosing were available, 5 experienced a decrease in CD3^−^CD4^+^ counts within weeks or months after initiation of OCS monotherapy. With the exception of P6 however, this effect was temporary and a subsequent rise in abnormal T cell counts was observed as OCS dosing was tapered. In the 5 other patients, CD3^−^CD4^+^ cells remained stable or even increased (P1) with OCS monotherapy. Interferon-α induced a significant reduction in CD3^−^CD4^+^ T cell counts in P1, P5, P10, and P25, but not in P12 whose eosinophil counts remained elevated as well. Neither fludarabine nor classical CHOP chemotherapy eradicated the abnormal T cell clone in P2.

## Discussion

In this retrospective observational study on the largest cohort of patients with CD3^−^CD4^+^ T cell-associated HES reported so far, we provide new insight on clinical manifestations, disease course and treatment responses of this rare disease, and further strengthen several previous observations as discussed below.

### Clinical Disease Features

Clinically, disease patterns in our cohort were heterogeneous and varied across a spectrum, from asymptomatic HE (HE_US_) to severe systemic treatment-refractory disease associated with significant disease- and treatment-induced morbidity and even mortality in some cases. Between these two extremities, the remaining patients presented cutaneous and soft tissue involvement of variable severity.

The association between CD3^−^CD4^+^ T cells and “HE_US_” has already been reported by others, with this lymphocyte subset being found in a significant proportion of patients with asymptomatic HE [3/8 HE_US_ patients in the NIH series (14)]. It should be noted however that among patients with CD3^−^CD4^+^ T cell associated HE, this particular clinical presentation is uncommon. Indeed, only 1/21 subjects in the French cohort (5) and 2/26 subjects in our cohort were classified as HE_US_. Four additional patients in our series were asymptomatic for more than a year after HE was first detected, then developed angioedema. Lymphocyte phenotyping should therefore definitely be included in the work-up of patients with asymptomatic HE, and secondary appearance of angioedema should raise suspicion of underlying L-HES even more.

The present series confirms the high prevalence of cutaneous symptoms in L-HES (20/24 or 83% of symptomatic patients) and their heterogeneity. Skin lesions and/or pruritus were actually the presenting symptom in most cases, so dermatologists are well-positioned to make an early diagnosis if they include L-HES in the differential diagnosis of skin disease associated with blood HE.

The second most prevalent symptom, present in 54% of the cohort including all 4 symptomatic patients without cutaneous involvement, is angioedema. Angioedema is generally symmetrical and may be relatively discreet, involving only the hands or part of the face, or it may be diffuse, involving the extremities and abdomen and resulting in significant weight-gain. The swelling is episodic, although eosinophil counts are constantly above 1.5 G/L even between flares in our series. Angioedema was associated with severe dermatitis in 3 cases, and increased serum IgM levels in roughly half as previously reported (5). That episodic angioedema is one of the classical clinical presentations of CD3^−^CD4^+^ T cell associated disease is further substantiated by the fact that these cells were detected in peripheral blood from all 4 subjects investigated in detail at the NIH (15), and 8/30 (27%) French patients with this condition (16). The central role of CD3^−^CD4^+^ T cells in pathogenesis is strongly supported by the fact that serum IL-5 levels precede peak eosinophil counts and symptomatic flares, although other cell types including eosinophils participate in the actual symptoms (15, 17).

Swelling of the extremities in subjects with CD3^−^CD4^+^ T cells may also be related to fasciitis and/or myositis. Indeed, several patients in our series reported episodic stiffness, pain, and induration of muscles, triggered by physical exertion in some cases. Although these patients had normal serum creatine kinase and troponin levels, fasciitis and/or myositis was confirmed by imaging (MRI, PET-FDG) or biopsy in four cases. This highly incapacitating complication of HES is under-recognized and may be confounded with angioedema. We therefore recommend assessment for fasciitis using MRI in presence of myalgia associated with painful muscle induration and swelling of the extremities. Another symptom that may lead patients to consult a rheumatologist is recurrent arthritis and/or tenosynovitis, observed in 3 patients in our series, and 4/21 patients in the French series. Lymphocyte phenotyping showed the presence of CD3^−^CD4^+^ cells in synovial fluid in two of our patients, and an identical TCR gene rearrangement to that observed in blood was found in a synovial biopsy from a French patient (9), establishing a direct role for these cells in synovial inflammation. Similar observations have been reported in patients with CD3^−^CD4^+^ T cells in the setting of angioimmunoblastic T cell lymphoma (18).

Other organs affected in more than one subject in our series include lungs (asthma and pulmonary infiltrates in two subjects each), secondary lymphoid organs, and the vascular system.

Lymphadenopathy is common (50 and 62% in ours and the French series, respectively), with effacement of lymph node architecture but sparing of the cortical sinus, presence of CD3^−^CD4^+^ T cells and an identical TCR gene rearrangement pattern as in peripheral blood (9), and increased metabolic activity. These characteristics may lead to a mistaken diagnosis of peripheral T cell lymphoma and initiation of overly aggressive treatment that may be fatal (patient P25 in our series and P3 in the French series) (9). We recommend utmost caution when interpreting findings on lymph node biopsies in this setting, and always refer them to histopathologists with expertise in T cell lymphoproliferative disorders to rule out underlying malignancy.

As for blood vessel involvement, Raynaud's phenomenon was the most frequent finding experienced by 6 subjects, with thrombosis of digital arteries and necrosis in one case. Occurrence of multiple arterial aneurysms in patients with CD3^−^CD4^+^ L-HES has been reported (5, 19), and we report a third case with a dissecting aneurysm herein, suggesting that large vessel involvement may be a rare but serious complication in this disorder. Myocardial involvement on the other hand is particularly uncommon in L-HES as can be inferred from the fact that none of the 47 patients from the combined Belgian and French cohorts were affected, despite prolonged and marked HE. In fact, only 2 patients with CD3^−^CD4^+^ associated HES and typical eosinophil-mediated cardiac damage have been reported to date (20, 21).

### Diagnostic Evaluation

Diagnostic assessment for L-HES is made by lymphocyte immunophenotyping and analysis of TCR gene rearrangement patterns by PCR to detect the clonal CD3^−^CD4^+^ T cells in peripheral blood. Using a specific lymphocyte phenotyping panel for patients with persistent HE followed by acquisition of a large number of CD4^+^ T cells and validation of results by an experienced biologist, we identified 11 subjects with 2% or less abnormal cells, 6 of whom had a supposedly normal phenotype at time of referral. We were able to confirm that these cells over-produce IL-5 in all cases. Hence, the proportion of patients with L-HES among those presenting with a HES is probably underestimated in many centers. Furthermore, we identified a patient whose clonal CD4 T cells were CD3-dim rather than CD3-negative. To our knowledge, this is the second such case reported so far (8).

Adaptation of the work-flow of lymphocyte phenotyping for patients with HES, is currently the most reliable approach for identifying patients with CD3^−^CD4^+^ T cell associated L-HES. Indeed, as shown in this study, none of the other tests available in routine are sufficiently sensitive or specific. The detection limit for clonality in peripheral blood using analysis of TCR gene rearrangement patterns by PCR is roughly 5%, explaining that none of the 11 patients with a small CD3^−^CD4^+^ subset in our series had a clonal TCR gene rearrangement pattern. Next generation sequencing does not currently seem to provide added sensitivity (unpublished observation), but further improvement of the molecular tools and/or of the algorithm used for clonality assessment may allow detection of specific and recurrent signals on a predominantly polyclonal background in the future. As for specificity, presence of an isolated clonal rearrangement in the setting of chronic HE is a common finding (22, 23). Whether the repeated observation of a clear-cut clonal population is indicative of T cell driven HE despite the absence of a detectable aberrant phenotype in peripheral blood remains to be determined. Other markers include mild to moderate elevations in serum LDH levels (observed in two thirds of our cohort), elevated serum IgE levels and/or polyclonal hypergammaglobulinemia (IgG, IgM) (each observed in just over half of our cohort, including 7/11 patients with small clones).

In a previous study, we showed that serum levels of CCL17/TARC were elevated in patients with evidence for IL-5-driven HES and suggested this could be a useful diagnostic biomarker (11). In this series, 92% (24/26) of patients had a significantly increased serum CCL17/TARC level (above 1,500 pg/ml) at some point during disease course, including all 11 patients with small clones. Serum CCL17/TARC is therefore a sensitive marker for CD3^−^CD4^+^ T cell associated L-HES, that fluctuates in concert with disease activity [e.g., during flares in subjects with EAE (15)]. This test is not routinely available, however, nor is it specific as suggested by a multicenter retrospective study showing elevated CCL17/TARC levels in 36% of HES patients without a clonal or aberrant T cell population (24).

### Disease Course

Disease course is difficult to delineate in rare conditions that are generally reported as case reports and small series. Constitution of this mono-centric patient cohort commenced 25 years ago, with detailed clinical reports enabling qualitative albeit retrospective analysis of patient outcome over a prolonged period of time. Median duration of follow-up since initial presentation with HE and symptoms (when present) in our series was 7.1 years, with 18 subjects followed for over 5 years and 23 for over 3 years.

One third of our cohort has not required maintenance therapy, either because they were asymptomatic (HE_US_, *n* = 2) or because disease manifestations were extremely mild and/or intermittent over a prolonged period of time (*n* = 7). Typically, the latter had either occasional urticaria, mild facial angioedema, and/or very limited cutaneous lesions. In two subjects with prolonged (>10-year) follow-up, disease manifestations and blood eosinophilia progressively decreased over time and finally disappeared despite persistence of a small population of CD3^−^CD4^+^ T cells.

The other two-thirds of our cohort had symptoms that warranted maintenance therapy. A subset of these patients (a third) presented with rapidly progressive constitutional and systemic symptoms in addition to pruritus, skin lesions, and/or angioedema, all requiring addition of a second line agent generally within months after onset because of disease progression despite OCS therapy. Although heavily treated, they continued to have symptoms and variable eosinophilia requiring frequent courses of high-dose OCS. Three of these patients died (two after development of lymphoma, and one after ASCT for treatment-refractory disease). The remaining patients presented with chronic cutaneous and/or soft tissue disease (angioedema, fasciitis) of variable severity, justifying addition of second-line agents in half.

Development of T cell lymphoma is an adverse outcome of particular interest in patients with CD3^−^CD4^+^ T cell associated HE. Combining our data with the French eosinophil network (9), it can be estimated that 10% (5/49) of patients progress to peripheral T cell lymphoma, namely angioimmunoblastic T cell lymphoma in 3/5 cases. The delay between initial presentation and lymphoma in these two cohorts varied from 4 to 15 years (mean 9 years), and no specific clinical or biological features at presentation were predictive of this outcome. Although a partial 6q deletion was observed prior to lymphoma development in one patient in our series (P2) (25) and in one French patient, 6q deletions may also be observed in L-HES in the absence of malignant progression (5). At time of lymphoma diagnosis, no major increases in CD3^−^CD4^+^ counts were observed compared to baseline levels in our series, whereas one French patient experienced a sharp rise in initially low counts at the time AITL was diagnosed. Lymphoma was suspected on the basis of rapid lymph node enlargement in all 3 of our patients and was confirmed by lymph node biopsy. In the French series, both patients with lymphoma had malignant cells in skin and bone marrow biopsies as well. As reported by others (9), our patients either failed to respond to, or died of complications of conventional chemotherapy (modified CHOP regimens). One patient in our series was cured with ASCT and is still alive.

Overall, our cohort illustrates the highly heterogeneous disease course in patients with CD3^−^CD4^+^ T cell associated HE, with roughly one third who experience little (if any) symptoms requiring little (if any) treatment and who may either remain stable over a prolonged period or may even improve spontaneously, while the other two thirds have debilitating clinical manifestations requiring long-term therapy, with significant disease and/or treatment-related morbidity in some, eventually progressing to lymphoma and/or death. At presentation, elevated serum IgM levels, but not CD3^−^CD4^+^ T cell or eosinophil counts, were associated with a more severe disease course. Over time, changes in CD3^−^CD4^+^ T cell counts paralleled disease activity in many cases. Indeed, sharp rises in abnormal T cells were observed in conjunction with worsening of symptoms in several patients, and conversely, counts dropped in several patients whose symptoms improved over time whether they were treated or not.

We therefore recommend repeated quantification of CD3^−^CD4^+^ cells during follow-up to assess for disease progression and adapt treatment accordingly. In patients treated with OCS, timing of blood sampling with regard to that of treatment absorption should be the same for serial measurements given that OCS has been shown to induce rebound morning lymphocytosis (26). This involves the major lymphocyte subsets (CD4, CD8, and CD19), but it remains unknown whether the clonal CD3^−^CD4^+^ T cells are similarly affected. The frequency of T cell phenotyping should be dictated by the severity of disease. We quantify CD3^−^CD4^+^ T cells at least twice a year, and more frequently in patients whose disease is uncontrolled or worsens. Given the absence of known risk factors or markers of malignant progression, we perform yearly FDG-PET scans to assess for appearance and/or progression of lymph node involvement that may indicate development of T cell lymphoma.

### Treatment Strategies

Oral CS remain the first-line treatment option for patients with HES except for those with chronic eosinophilic leukemia (27). Accordingly, all but one of the 17 patients requiring therapy to control disease manifestations in our cohort received OCS as initial treatment. None of our patients were CS-resistant, defined as absence of clinical and hematological response to 1 mg/kg/day PDN-equivalent. Although the dosing regimen required to observe a satisfactory response varied among patients, L-HES is a largely OCS-responsive disease, with the added benefit that treatment may decrease CD3^−^CD4^+^ cells counts in some cases (9). It should be noted however that disease was not cured with OCS alone in our series, and that the eventual reduction in abnormal T cell counts observed following initiation of therapy may be temporary.

In chronic inflammatory conditions like L-HES, once a response to OCS is observed, treatment is tapered to determine the minimally effective dose (MED) when possible. In our cohort, 60% of the subjects required a daily maintenance PDN-equivalent dose above 10 mg, a proportion similar to the French cohort (10/17) (5). All of these patients (*n* = 12) received second-line agents as detailed in the following paragraphs.

#### Interferon-Alpha

Our study confirms that among compounds currently available for HES, IFN-α is the most effective, with some degree of response observed in all 8 evaluable patients. There were three complete and five partial responders, and all were able to reduce background OCS treatment. Although IFN-α was initially used to treat HES patients with high myeloproliferative scores (28), it is also known to act on T cell polarization, and has specifically been to shown to reduce IL-5 production by clonal CD3^−^CD4^+^ cells *in vitro* (29). In the French series of 21 patients with L-HES, 7 out of 8 responded to IFN-α, making it the most effective commercialized treatment option in their experience (5). Another group reported a clear-cut reduction of absolute and relative CD3^−^CD4^+^ T cell counts in response to IFN-α (30) similar to our observations herein. Such observations, together with the fact that one patient in our cohort was cured of disease with IFN-α, mitigates the clinical relevance of our previous *in vitro* investigations showing that IFN-α prolongs the survival of these cells *in vitro* in a cytokine-free milieu (31).

Recombinant IFN-α is increasingly being replaced by its pegylated form that has a more convenient dosing regimen. Two patients in our series presented a complete response to a weekly dose of 180 μ with good clinical tolerance, but one had to stop treatment due to severe neutropenia. Another group has recently reported prolonged steroid-free remission in response to peg-IFN-α in 4/5 patients with CD3^−^CD4^+^ T cell associated L-HES ranging from 10 to 95 months, with significant reductions in abnormal T cell counts (32). However, the fifth patient described in more detail as a case report (33) experienced disease recurrence while on therapy within months, as observed in two patients in our series with recombinant IFN-α.

On the basis of these combined observations, IFN-α currently represents the most effective second-line treatment option for L-HES. Treatment generally reduces the abnormal T cell burden and may even be curative in rare instances. Pegylated-IFN-α should be preferred to the recombinant form, because its dosing regimen is more convenient. However, as previously reported in a large multicenter retrospective study on patients with HES (24), poor tolerance is common, accounting for half of treatment interruptions in our series. Secondary resistance to IFN-α also appears to limit the long-term benefit in some patients with L-HES.

#### Interleukin-5-Targeted Therapy

Monoclonal antibodies targeting IL-5 or its receptor are not approved for treatment of HES but have been available for selected patients through clinical trial participation and/or compassionate use (34) (NCT002446686 for mepolizumab, an anti-IL-5 antibody). Although the rationale for use in patients with L-HES is strong given the proven role played by IL-5 in eosinophilic expansion in this variant, we report a disappointing response to anti-IL-5 treatment in our series that is not well reflected by the broad criteria generally used to define a treatment response. Indeed, on the basis of an observed improvement of symptoms *and/or* reduction of eosinophil counts, all five patients in our series had a partial response to mepolizumab. However, the reality behind these figures is that none of the patients presented a complete clinical response despite high dosing regimens (monthly 700–750 mg IV infusions) and reduced eosinophil counts (3 complete, 2 partial hematological responses). Only two patients experienced partial symptomatic improvement with reduced intensity and frequency of angioedema flares, as reported by another group (35), although complaints related to fasciitis persisted. Symptoms (pruriginous skin lesions, angioedema, asthma, fasciitis, tenosynovitis) persisted in the three other patients, and dermatitis even worsened in two, with biopsies showing mixed eosinophilic and lymphocytic infiltrates. In the same line, one group reported complete absence of clinical response to mepolizumab in a patient with CD3^−^CD4^+^ HES despite normalization of blood eosinophilia (36), and investigators at the NIH reported that only 2 of the 6 L-HES patients receiving mepolizumab on a compassionate use basis presented a complete response allowing for a reduction in OCS dosing (37). Similarly, among 4 subjects with CD3^−^CD4^+^ T cells receiving benralizumab, a monoclonal anti-IL-5 receptor antibody, in the setting of a single-center pilot study, 3 relapsed within months after treatment initiation (38).

Overall, treatment directed against IL-5 or its receptor may not be the ideal strategy for patients with L-HES on the basis of the limited data presented above. However, we believe it is worthwhile to attempt treatment given the published evidence that IL-5/R targeting may benefit a subset of patients with CS-dependent CD3^−^CD4^+^ associated HES (5, 12, 35, 39, 40), our observation that angioedema may be attenuated by treatment, the excellent safety profile, and the absence of alternative well-tolerated treatment options. Given the high-level endogenous production of IL-5 in this HES variant, a high dosing regimen (e.g., 700 mg mepolizumab IV or 3 mg/kg reslizumab every 4 weeks) may be required to effectively neutralize this cytokine and should therefore be attempted before concluding that a patient is refractory. Biomarker sub-studies planned in the setting of multi-center clinical trials evaluating efficacy and safety of mepolizumab (NCT02836496) and benralizumab (NCT04191304) in HES should provide more definitive insight on clinical and hematological treatment responses specifically in patients with L-HES.

#### Immunosuppressive/Cytotoxic Compounds

None of the other immunosuppressive or cytotoxic compounds administered to our patients with refractory OCS-dependent disease, including cyclosporin A, hydroxyurea, methotrexate, cyclophosphamide, and fludarabine, had a significant favorable effect on disease. Among these agents, cyclosporin is the only drug reported by others to have some efficacy in L-HES (3/5 patients in the French series) (5). A recent single case report has shown a response to mycophenolic acid in a patient who had not responded to mycophenolate mofetil or peg-IFN-α (33). Alemtuzumab, a monoclonal anti-CD52 antibody able to target eosinophils in addition to the majority of leukocytes including lymphocytes, has been used anecdotally to treat CD3^−^CD4^+^ HES (21, 36) with an observed decrease in the CD3^−^CD4^+^ T cell count in one case. Besides its limited availability, use of alemtuzumab in this setting is not justified given significant treatment-related morbidity (41). Janus kinase (JAK) inhibitors represent a promising treatment option in selected patients with L-HES. Indeed, a gain-of-function STAT3 mutation and/or over-expression of STAT3 gene targets were demonstrated in 3 patients with CD3^−^CD4^+^ cells. On the basis of this observation, a small pilot study was undertaken, showing a favorable effect of tofacitinib on dermatitis in 2 patients with CD3^−^CD4^+^ L-HES (42). A phase 2 clinical trial has been designed to explore the efficacy of another JAK2 inhibitor, ruxolitinib, on a larger cohort of patients with HES including the lymphocytic variant (NCT03801434).

#### Allogeneic Stem Cell Transplantation

Allogeneic stem cell transplantation was performed in three patients in our series, to treat malignant lymphoma in one case, and as an ultimate strategy to control severe treatment-refractory non-malignant disease in the two others. The patient with lymphoma was cured. One patient with non-malignant disease died of ASCT-related complications, as previously reported for one patient in the French cohort (9), and the other had persistent disease. To our knowledge, there is only one report of a patient with non-malignant CD3^−^CD4^+^ T cell associated HES who was successfully treated with ASCT. He had an HLA-identical sibling and a reduced intensity conditioning regimen was used. The procedure resulted in disappearance of CD3^−^CD4^+^ T cells (20). Although currently the only means of possibly curing HES subjects with an aberrant T cell clone, ASCT should be reserved for carefully selected cases, namely those who develop malignant lymphoma, given the morbidity and mortality associated with this approach.

In conclusion, this retrospective and detailed longitudinal study of the largest single-center cohort of CD3^−^CD4^+^ T cell associated L-HES reported so far provides clinicians with relevant new data on patient presentation and outcome that should improve diagnosis, treatment and follow-up of this rare disorder (see Box 1). Specifically, we highlight certain clinical presentations that should heighten suspicion of L-HES including development of angioedema following initially asymptomatic HE, as well as fasciitis, a particularly painful, incapacitating and often undiagnosed complication of this disease. We report the small size of the abnormal T cell subset in a significant proportion of subjects, that is often overlooked during routine lymphocyte phenotyping, and show that changes in CD3^−^CD4^+^ cell counts over time in a given patient generally parallel changes in clinical disease activity. Our study underlines how difficult it can be to treat patients with CD3^−^CD4^+^ T cell L-HES who require high-dose OCS for disease control. Although IFN-α is currently the most effective second-line option actually curing disease in one patient in our series, poor tolerance and/or secondary resistance limit long-term benefit in the majority of cases. Therapy directed against IL-5 is in contrast extremely well-tolerated but the clinical response was disappointing in our series, despite a favorable effect on eosinophil counts. Overall, our study shows that CD3^−^CD4^+^ T cell L-HES is a heterogenous condition with regard to clinical manifestations, disease course, and treatment responses and its underscores the need for novel therapeutic options for the subset of patients with severe treatment-refractory disease. Future research efforts should be made toward understanding CD3^−^CD4^+^ T cell biology in order to develop new treatments that target primary pathogenic mechanisms.

Box 1 **Lessons learned on CD3-CD4+ T cell associated L-HES from this study.**

**Table:** 

**Novel findings**	**Recommendations**
**Clinical presentation**	
All symptomatic patients present with dermatitis, angioedema or both.	
Fasciitis is a common and debilitating complication that may be mistaken for angioedema.	In presence of episodic pain and stiffening in extremities, perform MRI (with gadolinium) and/or muscle biopsy to detect fasciitis.
**Diagnosis**	
A significant proportion of patients have very small subsets of CD3^−^CD4^+^ T cells that may be overlooked by routine lymphocyte phenotyping and TCR gene rearrangement studies.	Adapt flow cytometry methodology for patients being investigated for HES variants: use of an enlarged staining panel (including markers such as CD2, CD5, CD45RO, CD95) and acquisition of a large number of CD4+ T cells.
**Treatment**	
60% of oral corticosteroid-dependent patients need a daily maintenance dose above 10 mg PDN-equivalent for disease control.	
High serum IgM levels at presentation predict more severe disease requiring second-line therapy.	Consider introduction of a second-line agent early in disease course in patients with high serum IgM levels.
IFN-alpha is the most effective available second-line option, reducing both abnormal cell counts and the need for corticosteroid treatment. However, treatment often has to be interrupted due to poor tolerance and/or secondary resistance.	Pegylated IFN-alpha is the preferred currently available second-line agent.
Although anti-IL-5 antibodies lower eosinophil counts, clinical responses are disappointing even at high dosing regimens. The only disease manifestation that responded partially in our study was angioedema.	Anti-IL-5 treatment, if initiated, should be given at a high dosing regimen (e.g., 700 mg IV mepolizumab) before concluding it is ineffective.
**Disease course**	
Disease course is extremely heterogeneous.	
One third of patients have mild (if any) symptoms that do not warrant maintenance treatment, and that may even regress spontaneously over time.	Avoid (over-)treating patients whose clinical manifestations do not justify maintenance therapy, even if blood eosinophil counts are high. Prefer careful follow-up for occurrence of more serious complications.
One fifth of patients have severe progressive disease manifestations that are largely treatment-refractory.	Novel therapeutic options are desperately needed for this patient subset.
The remaining patients experience variable degrees of disease severity and need prolonged maintenance treatment. They suffer the combined adverse effects of chronic inflammatory disease and of therapeutic agents.	Adapt treatment to achieve the best benefit/risk ratio. Treatment dosing should be tailored to symptom control rather than blood eosinophil count.
Changes in CD3^−^CD4^+^ T cell counts over time are generally associated with fluctuations in disease activity.	An increase in CD3^−^CD4^+^ T cell counts should prompt investigations for disease progression (including lymphoma), and adaptation of treatment.
Roughly 10% of patients develop T cell lymphoma (estimated on a sample of almost 50 patients when combining this study with the French cohort). No disease features at presentation are predictive of this outcome. Progression of lymphadenopathy led to diagnosis in all 3 patients in our study.	Follow-up should include regular clinical assessment of lymphadenopathy and FDG-PET. ASCT should be considered early to treat T cell lymphoma arising in this setting as conventional chemotherapy is not effective.

## Data Availability Statement

The raw data supporting the conclusions of this article will be made available by the authors upon request, without undue reservation.

## Ethics Statement

This study was reviewed and approved by Comité d'Ethique Hospitalo-Facultaire Erasme-ULB. Written informed consent was obtained from the individual(s) for the publication of any potentially identifiable images or data included in this article.

## Author Contributions

CC, SV, and FR compiled the data from patient records and analyzed it. LS developed the flow cytometry protocol for sensitive detection of CD3^−^CD4^+^ T cells and analyzed all results. PH analyzed the TCR gene rearrangement patterns. A-LT retrieved, viewed and commented all biopsies. EC and FR recruited and followed all patients in the clinic. All authors agreed to the final version of this manuscript.

## Conflict of Interest

FR has received consultancy fees from GlaxoSmithKline, Astra Zeneca, and Knopp Biologicals regarding drug development for patients with hypereosinophilic syndromes. The remaining authors declare that the research was conducted in the absence of any commercial or financial relationships that could be construed as a potential conflict of interest. The reviewer BB declared a past co-authorship with one of the authors FR to the handling Editor.

## Abbreviations

CBC: Complete Blood Count
CEL: Chronic Eosinophilic Leukemia
CHOP: Cyclophosphamide Hydroxyldaunorubicin(Doxorubicin) Oncovin®(Vincristine) Prednisone
CRP: C Reactive Protein
EAE: Episodic Angioedema with Eosinophilia
ELISA: Enzyme-Linked Immunosorbent Assay
FIP1L1: Factor Interacting with PAPOLA and CPSF1
GM: geometric mean
HE: Hypereosinophilia
HES: Hypereosinophilic Syndrome
HEUS: Hypereosinophilia of Unknown Significance
IFN: Interferon
Ig: Immunoglobulin
L-HES: Lymphocytic Variant Hypereosinophilic Syndrome
LDH: Lactate Dehydrogenase
MED: Minimally Effective Dose
MRI: Magnetic Resonance Imaging
OCS: Oral Corticosteroid
PDGFRA: Platelet-Derived Growth Factor Receptor Alpha
PET: Positron Emission Tomography
R-CHOP: Rituximab-CHOP (see above)
TARC: Thymus and Activation-Regulated Chemokine
TCR: T Cell Receptor
WBC: White Blood Cells.
