# Supplementary material for: Eosinophilia Associated With CD3−CD4+ T Cells: Characterization and Outcome of a Single-Center Cohort of 26 Patients
Source: Front Immunol. 2020 Aug 11;11:1765. doi: 10.3389/fimmu.2020.01765 (PMC7432433; doi:10.3389/fimmu.2020.01765)
Supplement: Supplementary file 1 [file Data_Sheet_1.docx]

Eosinophilia associated with CD3-CD4+ T cells: characterization and outcome of a new cohort of 26 patients

Carpentier C^1†^, Verbanck S^1†^, Schandené L^2^, Heimann P^3^, Trepant AL^4^, Cogan E^1^, and Roufosse F^1,5*^.

**Supplementary material (Tables S1-S7 and Figure S1)**

**Supplementary tables**

Suppl Table S1. Flow cytometry antibody combinations for analysis of T cell phenotype in subjects with persistent HE.

| Tube | FITC | PE | PerCP-Cy5.5 | APC | APC-H7 |
| --- | --- | --- | --- | --- | --- |
| 1 | CD4 | FMO | CD3 | FMO | CD8 |
| 2 | CD4 | TCR-GD | CD3 | TCR-AB | CD8 |
| 3 | CD4 | HLA-DR | CD3 | CD2 | CD8 |
| 4 | CD4 | CD45RO | CD3 | CD45RA | CD8 |
| 5 | CD4 | CD25 | CD3 | CD62L | CD8 |
| 6 | CD4 | CD69 | CD3 | CD95 | CD8 |
| 7 | CD4 | CD27 | CD3 | CD7 | CD8 |
| 8 | CD4 | CD5 | CD3 | TCR-AB | CD8 |
| 9 | CD4 | CD28 | CD3 | CD57 | CD8 |

The CD3^-^CD4^+^ T cell subset can reliably be detected on the basis of the following characteristics:

- the level of CD4 expression is similar to the normal CD3^+^CD4^+^ T cells in the same tube
- 100% of the CD3^-^CD4^+^ T cells express membrane CD28, CD45RO and CD95
- the level of expression (mean fluorescence intensity) of CD2 and CD5 on CD3^-^CD4^+^ T cells is higher than on CD3^+^CD4^+^ cells
- a variable proportion of CD3^-^CD4^+^ T cells lack membrane expression of CD7 and/or CD27
- a variable proportion of CD3^-^CD4^+^ T cells express CD25 and/or HLA-DR.

Suppl Table 2. TCR gene rearrangement patterns and serum tryptase and TARC levels.

|  | TCR β/γ gene rearr pattern ^a^ | | | Tryptase ^b^ (μg/L) | TARC ^c^ (pg/ml) | |
| --- | --- | --- | --- | --- | --- | --- |
|  | Initial | Last | Interval (years) |  | Pre-Tx | Peak |
| P1 | + | NA |  | 13 ^e^ | 161032 | nd |
| P2 | + | + | 3 | 3.16 | 19249 | 82508 ^e^ |
| P3 | + | + | 22 | 2.58 ^e^ | 29600 | 44104 ^e^ |
| P4 | - | NA |  | 5.7 | 95600 | nd |
| P5 | + | (+) ^d^ | 10 | 3.48 ^e^ | nd | 50988 ^e^ |
| P6 | + | + | 14 | 2.02 | 3466 | 5242 ^e^ |
| P7 | - | (+) | 11 | 10.1 | 1407 | 3341 |
| P8 | (+) | (+) | 8 | 6.5 | 19618 | 22570 |
| P9 | (+) | + | 8.5 | 10.3 | 2980 | 41426 |
| P10 | - | (+) | 5 | 4 | nd | 422787 ^e^ |
| P11 | (+) | - | 6 | 4.62 | 1770 | 76449 |
| P12 | + | + | 5.5 | 4.56 ^e^ | nd | 123393 ^e^ |
| P13 | + | + | 4.5 | nd | nd | 5303 ^e^ |
| P14 | + | + | 2.5 | 5.34 | 6027 | 6027 |
| P15 | - | - | 3 | 7.43 | 10621 | 63318 |
| P16 | + | + | 3 | 4.58 | 2474 | 11089 |
| P17 | - | NA |  | 7.05 | 800 | 3565 |
| P18 | (+) | - | 2 | 6.4 | nd | 723 ^e^ |
| P19 | - | - | 1.5 | 5.08 ^e^ | nd | 7713 ^e^ |
| P20 | + | + | 1 | 15.71 | 73900 | 101000 |
| P21 | + | + | 0.5 | 4.05 | 1097 | nd |
| P22 | - | - | 0.5 | nd | 1750 | nd |
| P23 | - | NA |  | 11.5 ^e^ | nd | 2765 ^e^ |
| P24 | - | NA |  | 7.26 | nd | 4305 |
| P25 | + | + | 3 | 3.62 | nd | 4593 ^e^ |
| P26 | - | - | 1 | 12.6 | 10430 | 10430 |

^a^ +: clonal rearrangement, (+): small clonal peak on a polyclonal background, - : no clonal rearrangement pattern observed

^b^ normal value < 8.23 μg/L

^c^ normal value on a sample of 31 healthy controls: median 243 pg/ml (range 34-973)(unpublished personal observation)

^d^ at last control when CD3^-^CD4^+^ T cells still detectable

^e^ treatment ongoing at time of assessment

nd: not done

Suppl Table 3. Second-line agents administered to individual patients during chronic phase disease.

|  | IFNα | HU | Mepo | CSA | Other | Comments |
| --- | --- | --- | --- | --- | --- | --- |
| P1 | + | + |  | + | IFNγ,MTX | Lymphoma, Deceased |
| P2 | + |  |  |  | Fludara | Lymphoma, ASCT, cured |
| P3 | + (Peg) |  | + | + |  |  |
| P5 | + |  |  |  |  | Cured |
| P10 | + |  | + | + | IFNγ, IM, tacro, CPA | Lymphoma, Deceased |
| P11 |  |  | + |  |  |  |
| P12 | + | + | + | + | MTX, dupilumab |  |
| P13 | + (Peg) | + |  |  |  |  |
| P18 |  | + |  |  |  | JAK2 V617F mut |
| P23 | + (Peg) |  |  |  |  |  |
| P24 | + | + | + | + | IM, MMF, resli, CPA, IVIg, ASCT | Lost to FU |
| P25 | + |  |  |  | ASCT | Deceased < ASCT |

ASCT : allogeneic stem cell transplantation ; CPA : cyclophosphamide ; CSA : Cyclosporin A ;
Fludara : fludarabine ; FU : follow-up ; HU : hydroxyurea ; IFN : interferon ; IM : imatinib mesylate; IVIg : intravenous immunoglobulins ; Mepo : mepolizumab ; MMF : mycophenolate mofetil ; MTX : methotrexate ; Mut: mutation ; peg: pegylated ; resli : reslizumab (anti-IL-5) ; Tacro: tacrolimus

Suppl Table 4. Response to maintenance OCS in patients receiving at least 6 months therapy.

| Patient | OCS dose (mg, PDN-equiv) | | Disease course in terms of OCS administration | 2^nd^-line Tx |
| --- | --- | --- | --- | --- |
|  | Initial | MED |  |  |
| P1 | 30 | 50 | Partial response to PDN 30 mg - dose increased until a complete response was observed at 50 mg - attempts to taper below 50 mg early in disease course resulted in recurrence of eosinophilia and symptoms | Y |
| P2 | 40 | UNK (>10) | Dose rapidly tapered to PDN 10 mg because of poor tolerance - disease active at PDN 10 - PDN interrupted before MED could be determined (poor tolerance) - complete temporary response to short course of PDN 80 mg | Y |
| P3 | 20 | 12.5 | Disease completely controlled at 1 year with PDN 12.5 mg - dose decreased to 10 mg for many years with partial response and satisfactory risk/benefit ratio, and occasional flares requiring temporary dose escalation - MED increased after 18 years stable disease, with major recurrence of HE and symptoms responding only partially to PDN 20 mg | Y |
| P5 | 10 | 20 | *Episodic angioedema.* Symptoms and HE controlled with PDN 20 mg, but dose decreased to limit toxicity - alternate PDN 5/10 mg dosing allowed satisfactory symptom control despite persistent HE, with occasional flares requiring temporary dose escalation - frequency and severity of flares worsened over time with need for > 20 mg maintenance PDN 5 years after presentation | Y |
| P6 | 10 | 10 | Eosinophils normalized and marked clinical improvement at PDN 10 mg, with persistence of mild symptoms (a single patch of eczema) - over 15-year FU, progressive decrease in OCS dose without clinical progression, in parallel with progressive decrease in CD3-CD4+ T cell count - currently 2.5 mg maintenance PDN | N |
| P8 | 20 | 10 | Complete clinical and biological response at PDN 10 mg - maintenance dose reduced to 5 mg due to side effects of OCS - clinical remission maintained despite recurrence of HE | N |
| P10 | 20 | > 20 | *Episodic angioedema*. Partial clinical and biological response with PDN 20 mg - maintenance dose decreased to alternate 5/10 mg PDN due to psychiatric side effects, with persistent HE and regular flares requiring temporary dose escalation to PDN 30 mg | Y |
| P11 | 10 | UNK  (>10) | Partial response to PDN 10 mg - MED never achieved due to marked psychiatric intolerance to OCS - PDN stopped definitively after 8 months (poor tolerance) | Y |
| P12 | 80 | 30 | Complete initial response to PDN 80 mg - rapid dose tapering, with recurrence of HE and symptoms at 20 mg - complete response observed at PDN 30 mg early in disease course - over time, progressive increase in OCS requirement for clinical control, with recurrence of severe skin involvement at PDN 30 mg | Y |
| P13 | 40 | >25 | Complete initial response to PDN 40 mg - progressive dose tapering to 25 mg allowing symptom control despite mild persistent eosinophilia - symptom recurrence at PDN 20, MED required to normalize eosinophils unknown (higher than 25 mg) - over 5-year FU, progressive reduction of OCS dose required to maintain a complete clinical response, in parallel with progressive decrease in CD3-CD4+ T cell count - currently 10 mg maintenance PDN - mild persistent asymptomatic eosinophilia (< 1G/L) | Y |
| P14 | 80 | 5 | *Episodic angioedema.* Complete initial response to PDN 80 mg - dose tapered to 5 mg within 3 months, with no clinical recurrence since initiation of therapy - stabilized with 5 mg maintenance PDN for the past 4 years - mild persistent eosinophilia (>0.5-0.7 G/L) | N |
| P18 | 40 | UNK | OCS started 1 week after HU - rapid complete response to PDN 40 - dose tapered progressively over 6 months and stopped - no recurrence of HE or symptoms since withdrawal of OCS (FU 2.5 years) - HU pursued as monotherapy for JAK2-mutated thrombocytosis | Y^a^ |
| P19 | 10 | 7.5 | Complete initial response to alternate dosing PDN 5/10 mg - after 3 years stable dosing, recurrence of HE and urticaria requiring dose escalation - MED increased to 20 mg - currently clinically controlled with 15 mg, with persistent mild eosinophilia (< 1 G/L) | N |
| P21 | 40 | <10 | Complete initial response to PDN 40 mg - progressive dose tapering to PDN 10 mg with no recurrence of erythroderma - short FU (<1 year) - MED for clinical control unknown | N |
| P23 | 80 | 10 | *Episodic angioedema.* Complete initial response to 80 mg - rapid dose tapering - recurrence of HE and symptoms below 10 mg - short FU (<1 year) – introduction of second-line Tx to prevent long-term toxicity of OCS in this young patient | Y |
| P25 | 60 | >20 | *Episodic angioedema*. Complete initial response to 60 mg - rapid dose tapering - maintenance OCS dose at 20 mg, but frequent flares at this dose requiring temporary dose escalation to 30 mg | Y |

FU: follow-up; HE: hypereosinophilia; HU: hydroxyurea; MED minimally effective dose; OCS: oral corticosteroid; PDN: prednisone; Tx: treatment; UKN: unknown

^a^HU started prior to OCS for thrombocytosis.

Suppl. Table 5. Response to second line therapies.

|  | Duration | Max dose | Combined Tx | Oral CS dose reduction | Biol response | Clin response | Reason for interruption |
| --- | --- | --- | --- | --- | --- | --- | --- |
| IFN-alpha | | | | | | | |
| P1 | 29 mo | 42 MU/wk | OCS + Ø /CSA /IFN-γ/HU | Yes | Complete (11 mo) | Partial | Poor tolerance, efficacy temporary |
| P2 | 11 mo | 35 MU/wk | Ø | - | Partial | Complete (9 mo) | Poor tolerance, efficacy temporary |
| P3 | 4 mo | Peg IFN-α 180 μg/wk | OCS + CSA | Yes (CSA stop) | Complete | Complete | NA (ongoing) |
| P5 | 27 mo | 14 MU/wk | OCS | Yes | Complete | Complete | Cured of HES |
| P10 | 11 mo | 7 MU/wk | OCS | Yes | Complete | None | Lack of efficacy |
| P12 | 10 mo | 15 MU/wk | OCS + HU/Ø | Yes | None | Complete | Poor tolerance |
| P13^a^ | 3 wks | Peg IFN-α 45 μg/wk | OCS | - | - | - | Poor tolerance, Patient decision |
| P23 | 2 mo | Peg IFN-α 180 μg/wk | OCS | Yes | Complete | Complete | Adverse event (drug-related) |
| P24^b^ | 10 mo | 14 MU/wk | UNK | - | - | - | Poor tolerance |
| P25 | 29 mo | 28 MU/wk | OCS | Yes | Partial | Partial | Adverse event (not drug-related) |
| Hydroxyurea | | | | | | | |
| P1 | 3 mo | 2 gr | OCS + IFN-α | No | None | None | Lack of efficacy |
| P12 | 16 mo | 2 gr | OCS + IFN-α/ Ø | Yes | Partial | Complete | Poor tolerance |
| P13^a^ | 1 mo | 0,5 gr | OCS | - | - | - | Patient decision |
| P18^a^ | 36 mo | 0,5 gr | Ø / OCS / Ø | - | - | - | NA (continued for thrombocytosis) |
| P24^b^ | 18 mo | 2 gr | OCS | - | - | - | - |
| Mepolizumab (compassionate use, MHE104317, NCT002446686) | | | | | | | |
| P3 | 10 mo | 700 mg IV | OCS | No | Partial | None | Lack of efficacy |
| P10 | 6 mo | 750 mg IV | OCS | No | Complete | Partial | SAE (Lymphoma) |
| P11 | 7 mo | 700 mg IV | Ø | - | Complete | Partial | NA (ongoing) |
| P12 | 8 mo | 700 mg IV | OCS | No | Complete | None | Lack of efficacy |
| P24 | once | 750 mg IV | Ø | - | Partial | None | Lack of efficacy |
| Cyclosporin | | | | | | | |
| P1^c^ | 5 mo | 400 mg | OCS + IFN-α | No | - | - | No effect on T cells |
| P3^c^ | 3 mo | 250 mg | OCS + Peg-IFN-α | Yes | - | - | Poor tolerance/Efficacy of IFN-α |
| P10 | 2.5 wks | 200 mg | Ø | - | None | None | Lack of efficacy |
| P12^c^ | 4 mo | 200 mg | OCS + Mepo 300 | No | - | None | Lack of efficacy |
| P24^b^ | 3 mo | UNK | UNK | - | - | - | - |

^a^ Dosing/duration not sufficient to evaluate treatment response.

^b^ Insufficient data collection to analyse response (patient received treatment in another center).

^c^ No added response to concomitant HES medications, and treatment interrupted due to poor tolerance (individual biological and clinical responses could not be evaluated because of overlap with other therapies)

Ø: none (concomitant treatment), CSA: cyclosporin A, gr: gram, HU: hydroxyurea, IFN: interferon, IV: intravenous, mepo: mepolizumab, mg: milligram, mo: month, MU: million units, NA: not applicable, OCS: oral corticosteroid, Peg: pegylated, SAE: serious adverse event, UNK or - : unknown, wk: week

Suppl. Table 6. Detailed disease course over time.

|  | Presentation | Disease course | - Outcome - Current Tx |
| --- | --- | --- | --- |
| P1 | Rapidly progressive severe systemic disease | Early initiation of maintenance OCS: requirement for continuous high-dose Tx; combination Tx throughout disease course; numerous relapses despite Tx; progression to anaplastic null cell lymphoma 4 yrs after first symptoms; Tx: splenectomy, dexamethasone, cyclophosphamide | - Death (septicemia during Tx for lymphoma) - NA |
| P2 | HEUS | Asymptomatic HE for 3 years. Development of slowly progressive, chronic, predominantly cutaneous disease; extension of eczema over several years and development of tenosynovitis eventually requiring systemic Tx; marked intolerance to Tx with interruptions leading symptom rebound; appearance of subcutaneous nodules and benign lymphadenopathy in head and neck region; transient response to fludarabine; rapid recurrence of symptoms (eczema, subcutaneous nodules, lymphadenopathy) leading to diagnosis of PTCL-NOS 15 years after first symptoms; Tx: modified CHOP (refractory) followed by ASCT | - Alive, in remission with 17-yr FU after treatment of lymphoma with ASCT - None |
| P3 | Rapidly progressive severe systemic disease | Early initiation of maintenance OCS, with excellent response; partial remission with stable OCS dose for 18 yrs, and occasional flares (pruriginous papules, angioedema, and/or asthma) requiring transient dose escalation; after 18 yrs, disease progression in parallel with increased CD3-CD4+ counts requiring increased OCS dose; initiation of 2^nd^ line Tx | - Excellent response to Peg-IFNα (4-mo FU) with regression of HE and symptoms and OCS-sparing - PDN 5 + Peg IFNα 180 microgr |
| P4 | HEUS | Asymptomatic HE for 2 years; development of intermittent, mild disease (urticaria); maintenance Tx not justified; progressive decrease in frequency of urticaria over the years in parallel with spontaneous decline in eosinophil and CD3-CD4+ cell counts | - Spontaneous biological and clinical remission 11 yrs after diagnosis, with 8-yr FU - None |
| P5 | Progressive recurrent episodic angioedema | Initiation of OCS 3 years after first symptoms; initial symptomatic improvement with regression of angioedema; pursuit of low-dose maintenance Tx with persistent blood HE and occasional flares requiring transient dose escalation; progressive increase in frequency and severity of flares over 5 years, requiring increased maintenance OCS-dosing; introduction of 2^nd^ line Tx with IFN-α; progressive decrease then disappearance of CD3-CD4+ T cells | - Cured: disappearance of CD3-CD4+ cells 2 yrs after initiation of IFN-α; no disease recurrence despite Tx interruption with 14-yr FU - None |
| P6 | Slowly progressive, chronic, cutaneous disease | Progressive extension of eczema over 13-yr period; introduction of OCS with low maintenance dose and excellent response; attempts to taper OCS resulting in symptom recurrence during 10 yrs; over the past 3 yrs, successful OCS tapering in parallel with a progressive decline in CD3-CD4+ cell counts | - Disappearance of eczema and eosinophilia with low-dose OCS - PDN 2.5mg |
| P7 | Intermittent, mild disease (urticaria and facial angioedema) | Maintenance Tx not justified; progressive decrease in blood eosinophilia and frequency of symptoms over time with stable (low) CD3-CD4+ T cell counts | - Spontaneous biological and clinical remission 11 yrs after diagnosis, with 2-yr FU - None |
| P8 | HEUS | Asymptomatic HE for 4 yrs; appearance of mild episodic facial angioedema; 10 yrs later, appearance of pruritus, myalgia, Raynaud’s; initiation of low-dose OCS with excellent clinical response | - Asymptomatic with low-dose OCS, despite persistent HE, with 9-yr FU - PDN 5 |
| P9 | Intermittent, mild disease (sparse bullous lesions) | Single bullous skin lesion with spontaneous resolution; 8-yr asymptomatic period with progressive rise in eosinophil counts; recurrence of several bullous lesions during 3 yrs; spontaneous prolonged remission despite absence of Tx  (development of Sjögren’s syndrome, possibly accounting for more recent development of Raynaud’s and mild tenosynovitis) | - no recurrence of skin lesions with 8-yr FU, despite persistent HE - None |
| P10 | Rapidly progressive systemic disease, severe episodic angioedema | Early initiation of maintenance OCS, with partial response and requirement for dose escalation during flares; rapid increase in frequency of relapses justifying initiation of 2^nd^ line Tx; refractory or partial response to various agents; development of AITL presenting as a single enlarged cervical lymph node 6 yrs after presentation; Tx: R-CHOP; disease progression despite Tx | - Death (palliative care, infectious complications of chemotherapy for lymphoma) - NA |
| P11 | Slowly progressive chronic disease (fasciitis, episodic angioedema and urticaria) | Initiation of maintenance OCS 2 yrs after presentation with good response; interruption of OCS after 7 months due to poor tolerance; no Tx for 4-yr period with stable incapacitating symptoms and HE; initiation of 2^nd^ line Tx with partial response | - Stable (moderate) intensity and frequency of symptoms since presentation, with 8-yr FU - Mepolizumab 700 mg/mo IV |
| P12 | 2 step presentation:  1. Intermittent, mild disease (urticaria and facial angioedema)  2. Progressive systemic disease | Episodic mild facial angioedema and rash over 3 yr-period with HE; spontaneous resolution with 6-yr asymptomatic period and normal eosinophilia; recurrence of HE and symptoms (episodic angioedema, fasciitis, fever) with progressive worsening over a 2-yr period; initiation of high-dose OCS with complete response, but recurrence of disease and HE while tapering Tx; refractory or intolerant to various 2^nd^ line agents over a 3 yr-period; marked worsening of cutaneous lesions despite continuous high-dose OCS in parallel with rapid increase in CD3-CD4+ counts | - Severe progressive and Tx-refractory disease despite high-dose OCS - PDN 25 + Dupilumab (4-wk FU) |
| P13 | Rapidly progressive systemic disease | Early initiation of maintenance OCS with rapid clinical and biological improvement; attempts to taper PDN dose below 30 mg unsuccessful; initiation of 2^nd^ line Tx (efficacy unknown due to patient refusal to pursue Tx due to side effects); progressive decrease of the MED OCS dose over a 3-yr period | - Progressive improvement over time with continuous OCS Tx, and reduction of MED dose of PDN over 3 yrs - PDN 10 mg |
| P14 | HEUS | Asymptomatic HE for 2 yrs; rapidly development of angioedema; initiation of maintenance OCS with rapid disappearance of symptoms and HE; OCS dose tapered down to PDN 5 over 3 mo; in remission with 4-yr FU | - Disappearance of angioedema and HE with OCS; no recurrence since initiation of Tx - PDN 5 mg |
| P15 | HEUS | Asymptomatic HE for 1.5-2 yrs; development of episodic mild facial and hand angioedema; complete rapid response to short course of low-dose OCS; since then, recurrent episodes treated with low-dose OCS for 3-5 days; progressive increase in the frequency of episodes during 3.5-yr FU | - Frequency of episodes mild facial angioedema increasing progressively - No maintenance Tx; short courses low-dose OCS for flares |
| P16 | Slowly progressive, mild chronic cutaneous disease | Intermittent mild urticaria since 9 yrs; fixed localized patch of eczema, with very slow increase in diameter over 5 yrs | - Slow progression of cutaneous symptoms; stable eosinophilia (1-1.5 G/L) - None |
| P17 | Stable mild chronic disease | Stable mild cholestasis since 10 yrs without fibrosis; stable fixed patch of eczema since 6 yrs | - Stable disease without Tx with 10-yr FU - None |
| P18 | Progressive cutaneous disease | Development of erythroderma over 6-mo period; rapid resolution of symptoms and HE with OCS, tapered down and stopped after 6 mo; 2-yr FU with HU alone (initiated to treat JAK2-mutated thrombocytosis) | - No recurrence with maintenance HU - HU 0.5-1 gr |
| P19 | Slowly progressive chronic predominantly cutaneous disease | Fortuitous detection of asymptomatic eosinophilic esophagitis treated with OCS for 6 wks; development of pruritis and urticaria 9 mo after discovery of HE; initiation of maintenance OCS with complete response; stable maintenance dose for 3.5 yrs; progression of disease with recurrence of urticaria and HE despite stable OCS dosing; increase in OCS dose | - Asymptomatic residual eosinophilia (0.5-1 G/L) with maintenance OCS - PDN 15 |
| P20 | Slowly progressive chronic cutaneous disease | Initially mild cutaneous symptoms with marked HE; progressive extension of skin involvement over 1.5 yrs | - Progression of cutaneous symptoms - None (considering initiation of OCS) |
| P21 | Progressive chronic cutaneous disease | Development of erythroderma over 6-mo period; initiation of maintenance OCS after 2 yrs; rapid resolution of symptoms; progressive tapering of OCS dose to 10 mg; reappearance of a single localized patch of dermatitis | - Partial clinical remission with stable maintenance OCS with 1-yr FU - PDN 10 |
| P22 | HEUS | No symptoms despite marked HE for at least 8 yrs | - HEUS |
| P23 | HEUS | Development of angioedema (hands and calves) 17 months after fortuitous discovery of HE; early initiation of maintenance OCS with rapid response; recurrence of HE and symptoms during tapering; initiation of 2^nd^ line Tx (Peg-IFN) 6 mo after OCS, with complete response but marked toxicity (neutropenia) leading to cessation; OCS monotherapy pursued; short FU (< 1 yr) | - Asymptomatic with maintenance OCS - PDN 10 |
| P24 | UKN (Presentation in another center) | UKN | - Lost to FU |
| P25 | Rapidly progressive systemic disease | Progressive extension of skin rash over 1 yr followed by rapid development of diffuse angioedema; initiation of OCS with rapid clinical and biological response; requirement of at least 20 mg PDN to prevent flares but dose often temporarily reduced below this threshold to prevent toxicity; frequent courses of higher-dose OCS; initiation of 2^nd^ line (Peg-IFN) allowing for reduction of OCS MED during 2-yr period; Peg-IFN interrupted due to AE (severe systemic EBV infection with marked lymphocytosis); recurrence of Tx-refractory disease; Tx: ASCT | - Death (infectious complications of ASCT for refractory (non-malignant) disease - NA |
| P26 | HEUS | No symptoms despite marked HE for at least 6 yrs | - HEUS |

AITL: angioimmunoblastic T cell lymphoma; ASCT: allogeneic stem cell transplantation; FU: follow-up; HE: hypereosinophilia; HEUS: hypereosinophilia of undetermined significance; HU: hydroxyurea; IV: intravenous; MED: minimally effective dose; mo: month; Peg: pegylated; PTCL-NOS: peripheral T cell lymphoma not otherwise specified; (R-)CHOP: (Rituximab) Cyclophosphamide Hydroxyldaunorubicin(Doxorubicin) Oncovin®(Vincristine) Prednisone; Tx: treatment; UNK: unknown; yrs: years

Suppl. Table 7. Evolution of CD3^-^CD4^+^ T cell counts over time in patients with at least 2 lymphocyte immunophenotyping studies.

|  | Presentation  abs (% lymphocytes) | Last control  abs (% lymphocytes) | Interval (months) |
| --- | --- | --- | --- |
| P1 | 631 (43%) | 615 (28%) ^a^ | 28 |
| P2 | 3000 (71%) | 2930 (73.5%)^b^ | 70 |
| P3 | 1717 (60%) | 2800 (69.3%) | 269 |
| P4 | 224 (10%) | 20 (0.98%) | 228 |
| P5 | 1728 (30%)^c^ | 0 | 212 |
| P6 | 115 (8.4%) | 23 (1.81%) | 169 |
| P7 | 10 (0.5%) | 13 (0.7%) | 135 |
| P8 | 34 (1.9%) | 27 (1.54%) | 111 |
| P9 | 138 (7%) | 106 (6.52%) | 101 |
| P10 | 34 (2%) | 60 (7.75%)^b^ | 59 |
| P11 | 10 (0.7%) | 17 (1.3%) | 75 |
| P12 | 559 (23%) | 2458 (67.3%) | 76 |
| P13 | 1148 (29%)^c^ | 167 (8.53%) | 53 |
| P14 | 1420 (71%) | 916 (65%) | 47 |
| P15 | 26 (1.2%) | 66 (2.78%) | 35 |
| P16 | 57 (7.4%) | 56 (8.24%) | 32 |
| P17 | 14 (0.46%) | 15 (0.5%) | 14 |
| P18 | 161 (13%) | 7 (0.63%) | 31 |
| P19 | 2 (0.15%)^c^ | 13 (0.57%) | 17 |
| P20 | 66 (2.7%) | 85 (3.13%) | 12 |
| P21 | 343 (22%) | 256 (15.14%) | 10 |
| P25 | 2709 (57%) | 2000 (15.6%) | 86 |
| P26 | 30 (1%) | 25 (1.41%) | 15 |

CD3-CD4+ T cell counts are shown at presentation and at the most recent available assessment at time of data collection (June 2019). Results are expressed as absolute counts, and percent total lymphocytes. The interval between these 2 measurements is expressed in months.

^a^ value at last control in our center (moved to foreign country) before development of lymphoma

^b^ value at time of diagnosis of T cell lymphoma

^c^ oral CS at time of first detection of CD3-CD4+ cells

Green : at least 50% decrease in absolute count

Red: at least 50% increase in absolute count: patients with very low baseline counts are shown in a lighter shade

**Supplementary figures.**

Suppl. Figure S1. CD3 and TCR Vβ4 chain expression by gated CD4 T cells in P25.

**Vβ4**

**CD3**


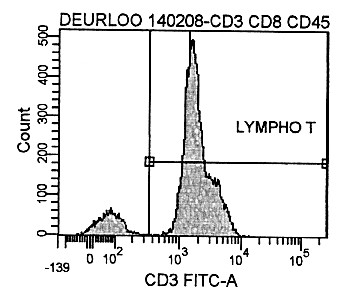


Legend to supplementary figure.

Figure S1.

CD3 and TCR Vβ4 expression are shown on gated CD4-positive lymphocytes.

Homogeneously decreased staining intensity for surface CD3 was observed on a significant proportion of CD4 T cells (T cell population shown left of the red line).

The TCR Vb4 sub-family was expanded (roughly 85% of CD4 T cells).
